# Serpin A1 Cargo Within Tumour‐Derived Extracellular Vesicles Supports Pro‐Tumoural Bias of Neutrophils in Cancer

**DOI:** 10.1002/jev2.70385

**Published:** 2026-11-02

**Authors:** Maksim Domnich, Ekaterina Pylaeva, Elena Siakaeva, Nastassia Kabankova, Agnieszka Bedzinska, Damian Sojka, Aneta Zebrowska, Marta Gawin, Maren Soldierer, Yaping Yu, Malwina Rist, Daniel Fochtman, Bernd Giebel, Iris Helfrich, Ilona Thiel, Basant Kumar Thakur, Cornelius H. L. Kürten, Helmut Hanenberg, Sonja Ludwig, Piotr Widlak, Stephan Lang, Monika Pietrowska, Jadwiga Jablonska

**Affiliations:** ^1^ Department of Otorhinolaryngology, University Hospital Essen University of Duisburg‐Essen Essen Germany; ^2^ Department of Dermatology and Allergy LMU Hospital Ludwig‐Maximilians‐University Munich Germany; ^3^ German Cancer Consortium (DKTK) Partner Site Düsseldorf/Essen Essen Germany; ^4^ Maria Sklodowska‐Curie National Research Institute of Oncology Gliwice Branch Poland; ^5^ Department of Pediatrics III University Children's Hospital of Essen University of Duisburg‐Essen Essen Germany; ^6^ Institute for Transfusion Medicine University Hospital Essen University of Duisburg‐Essen Essen Germany; ^7^ German Cancer Consortium (DKTK), Partner Site Munich Munich Germany; ^8^ Exosome Research Lab (ERL), Department of Gastroenterology Hepatology and Transplantation Medicine University of Duisburg‐Essen Essen Germany; ^9^ Department of Otorhinolaryngology Head and Neck Surgery University Hospital Mannheim Medical Faculty Mannheim University Heidelberg Mannheim Germany; ^10^ Department of Radiology Medical University of Gdansk Gdańsk Poland

**Keywords:** arginase 1, epithelial‐to‐mesenchymal transition (EMT), exosomes, head and neck cancer (HNC), immunosuppression, neutrophils, proteome, serpin A1, small extracellular vesicles (sEVs), STAT3

## Abstract

Tumour‐derived extracellular vesicles (EVs) or exosomes are critical mediators of the cancer‐immune system crosstalk, yet the mechanisms by which they reprogram neutrophil function in vivo remain insufficiently characterised. Here, we identify the serine protease inhibitor Serpin A1 as a predominant cargo component of small EVs (sEVs) released by head and neck cancer cels (HNC) and show that this molecule is both necessary and sufficient to confer a stable pro‐tumoural programming of neutrophils. Uptake of Serpin A1‐enriched sEVs elicits extensive proteomic remodelling in neutrophils including activation of STAT3 signalling, leading to sustained induction of PD‐L1, increased Arginase 1 production and prolonged neutrophil lifespan. Functionally, these reprogrammed neutrophils acquire a capacity to drive epithelial‐mesenchymal transition in tumour cells, and to impose exhaustion of T cells via metabolic restriction and arginine depletion, leading to their reduced cytotoxicity. Genetic ablation of Serpin A1 from tumour‐derived sEVs or pharmacologic inhibition of serpin‐STAT3 axis restores neutrophil anti‐tumour functions. Collectively, these findings delineate a Serpin A1‐STAT3/Arginase 1 as an important signalling pathway through which tumour‐derived sEVs promote neutrophil‐dependent immune suppression and tumour progression, and highlight EV‐associated serpins as potential targets for future therapeutic intervention in cancer.

## Graphical abstract

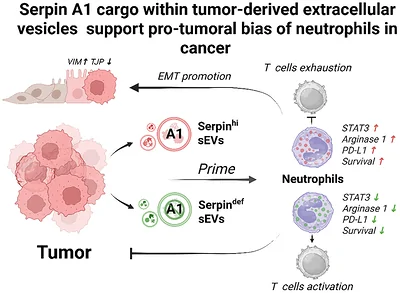

## 1 Introduction

Neutrophils have emerged as critical regulators of tumour progression, with their phenotype and function being extensively shaped by tumour‐derived cues (Jablonska et al. 2010; Fridlender et al. 2009). In patients with solid malignancies, including head and neck cancer (HNC), neutrophil counts in blood and tumour tissue are frequently increased and correlate with adverse clinical outcomes (Pylaeva et al. 2022; Trellakis et al. 2011; Albrengues et al. 2018; Anselmi et al. 2022), reflecting a shift from early anti‐tumoural activity toward a pro‐tumoural, immunosuppressive state as advanced disease (Andzinski et al. 2016). Although cytokines, chemokines and growth factors have been implicated in driving this functional transition (Granot and Jablonska 2015), the mechanisms that imprint a stable, pro‐tumourigenic program on neutrophils in vivo remain poorly defined.

Small extracellular vesicles (sEVs) or exosomes released by tumour cells have recently been recognised as potent vectors of a bioactive cargo capable of reprogramming immune cells (van Niel et al. 2018; Tengler et al. 2023). Tumour‐derived sEVs have been shown to modulate dendritic cell function, suppress T cell proliferation and alter NK cell activity, highlighting their central role in tumour‐immune cell communication (Zitvogel et al. 1998; Skokos et al. 2003). However, how tumour‐derived sEVs shape neutrophil phenotype, lifespan and functional polarisation, and which specific vesicular cargo components mediate these effects, are not yet understood.

Serine protease inhibitors (serpins) constitute a highly abundant class of secreted proteins in plasma with established roles in inflammation, tissue remodelling and cancer biology, yet their contribution as defined cargo within tumour‐derived sEVs has not been systematically explored (Sanrattana et al. 2019; Yuan et al. 2021; Valiente et al. 2014; Kuai et al. 2023).

Here, we investigate the role of tumour‐derived sEVs in neutrophil reprogramming during cancer progression and identify Serpin A1 as a key cargo component in these vesicles, driving the activation of the STAT3/Arginase 1‐axis in neutrophils and resulting in their anti‐tumoural reprogramming.

## 2 Materials and Methods

### 2.1 Patients

Peripheral venous blood specimens were collected from patients with head and neck cancer (HNC) (45 individuals) treated at the University Hospital Essen and healthy volunteers (10 individuals) from 2017 to 2019. The clinical characteristics of the enrolled participants are presented in Figure 1A,B and Figure S1. The patients were scheduled for surgical tumour resection and consented to blood collection for research purposes at the Otorhinolaryngology, Head and Neck Surgery Department of the University Hospital of Duisburg‐Essen, Essen, Germany. Patients with other or unknown histological types of tumours or cancer with unknown primary origin (CUP), tumours associated with specific risk factors like the nasopharyngeal squamous cell carcinoma, which is associated with Epstein‐Barr virus, as well as patients who received any cancer‐associated therapies before the study, were omitted. Informed consent was received from all individuals.

**FIGURE 1:**
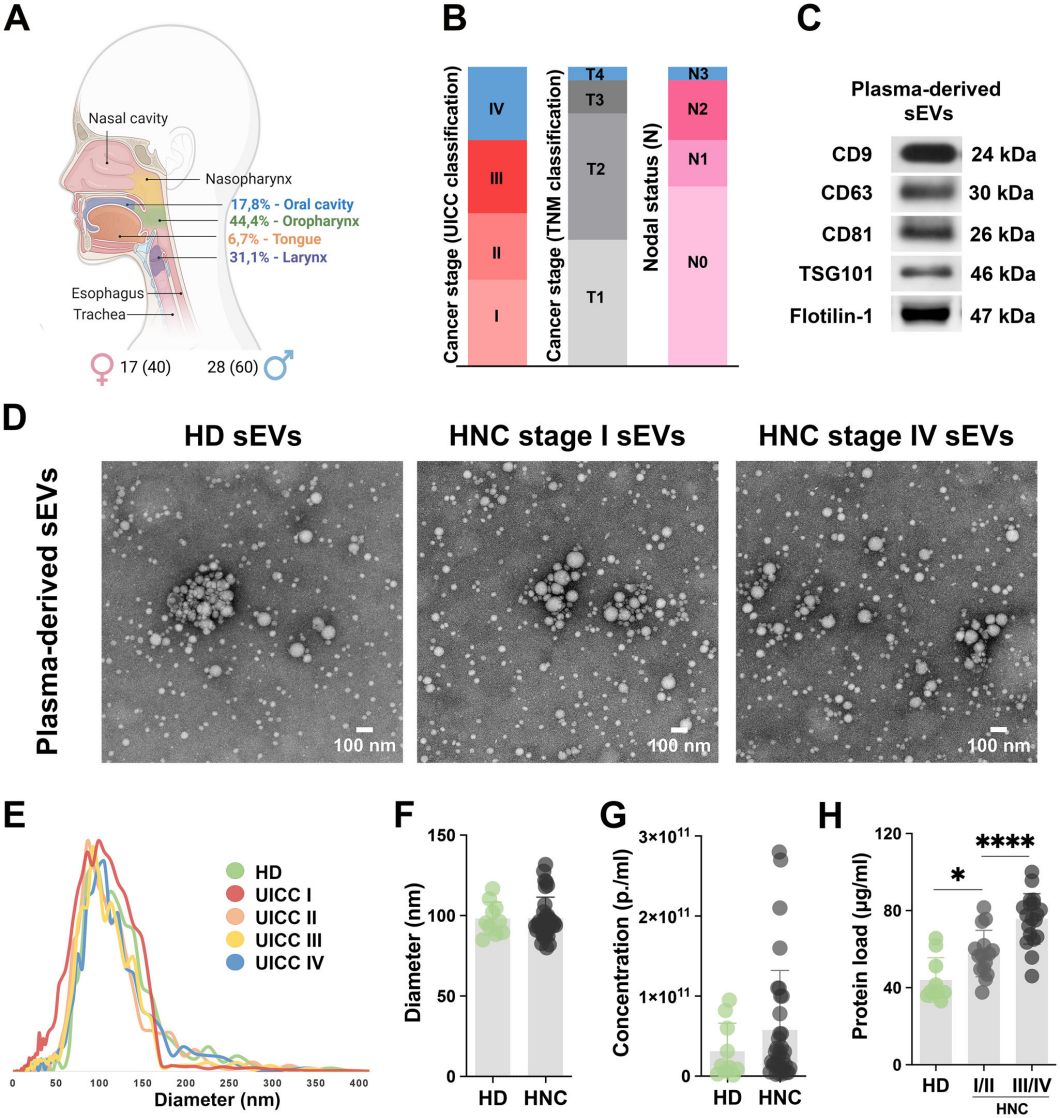
Patient‐derived sEV samples contain higher particle numbers than those of HD without revealing any size differences. (A) Plasma samples were collected from 10 healthy individuals (five males and five females, with a mean age of 38 years) and 45 patients (28 males, 60% and 17 females, 40%, with a mean age of 63 years). Primary tumour sites were oropharynx (44.4%) oral cavity (17.8%), including tongue (6.7%) and larynx (31.1%). (B) The patients were classified using the eighth edition of the Union for International Cancer Control (UICC) guidelines according to UICC stage I‐IV head and neck squamous cell carcinoma. Thirteen (28.8%) patients were UICC I, 10 (22.2%) UICC II, 11 (24.5%) UICC III and 11 (24.5%) UICC IV. (C) Using Western blotting, the presence of common sEVs markers such as CD9, CD81, CD63, TSG101 and Flotillin‐1 was determined in plasma‐derived (pd‐) sEVs from the plasma samples of the study participants. (D) Representative TEM micrographs of pd‐sEVs show intact vesicles ranging in size from 50 to 150 nm, consistent with NTA data. Scale bar 100 nm. (E) Nanoparticle tracking analysis (NTA) of sEVs enriched from plasma samples of HD and patients with low (UICC I/II) and high advanced (UICC III/IV) stages of HNC reveal similar mean sizes (∼100 nm) (E, F) and diversified concentrations (particles/mL) (G) of pd‐sEVs. (H) In comparison to cancer patients, healthy donors had significantly lower levels of total protein concentration (HD = 44 µg/mL, UICC I/II = 57.3 µg/mL, UICC III/IV 75.7 µg/mL). Scale bar 100 nm. For the comparison of two independent groups, the Mann‐Whitney U test was used. Data are shown as individual values with a median. *p < 0.05, ****p < 0.0001.

### 2.2 Isolation of Extracellular Vesicles From Human Plasma

Peripheral blood was drawn into sodium citrate anticoagulant S‐Monovette (Sarstedt, cat# 02.1067.001), centrifuged at 1500 × *g* at 20°C for 10 min, plasma was collected and aliquoted in 1‐mL tubes, and stored at –80°C until usage. Either using fresh plasma or after thawing stored plasma, samples were differentially centrifuged at 2000 × *g* for 10 min at 20°C, followed by centrifugation at 14,000 × *g* for 30 min at 4°C to remove large microvesicles and apoptotic bodies. Then, plasma was ultrafiltrated using a 0.22‐µm filter (Millex, cat# SLGP033RS). Next, the Econo‐Pac chromatography columns (BioRad, cat# 7321010) were filled with Sepharose CL‐2B up to the mark 10 mL (Cytiva, cat# 17‐0140‐01). Separation of plasma constituents was realised by application of consecutive 1 mL portions of PBS. The fourth fraction (F4), enriched in small extracellular vesicles, was collected for further investigation.

### 2.3 Cell Lines

UT‐SCC‐50: Human laryngeal squamous cell carcinoma UT‐SCC‐50 (p53‐frameshift, c.920del73/p14 ‐ No transcript/HPV16 E6/E7−), SCC50 in short, was obtained free of charge from the University Hospital Ulm (Hoffmann et al. 2008; Lin et al. 2007). UM‐SCC‐10a: Human squamous cell carcinoma of the larynx UM‐SCC‐10a (p53 ‐ missense, p.245Gly→Cys/p14 ‐ frameshift, c.388delC/HPV16 E6/E7−), SCC10a in short, was kindly provided by professor Thomas E. Carey (PhD, Director, Laboratory of Head and Neck Cancer Biology, Department of Otolaryngology/Head and Neck Surgery University of Michigan, Ann Arbor, USA) (Bradford et al. 1991; Baker 1985).

### 2.4 Knockout of Serpin A1 in UM‐SCC10a Cells

For the Serpin A1 CRISPR/Cas9 knockout construct, DNA oligonucleotides for the gRNAs (CTATGATCTGAAGAGCGTCC for SERPIN A1), as well as a control gRNA targeting chicken beta‐actin, were cloned into the lentiviral CRISPR/Cas9 vector LentiCRISPRv2 (Addgene, cat# 52961) using BsmBI restriction enzyme sites as described (Virts et al. 2015). VSV‐G‐pseudotyped replication‐deficient infectious lentiviral particles were produced in HEK293T cells as previously described (Wiek et al. 2015). After filtration through a 0.45 µm filter, the lentiviral particles were used freshly for transduction of UM‐SCC10a cells. After 24 h, the viral supernatant was replaced with a fresh cell culture medium. Three days after transduction, cells were selected with 1 µg/mL puromycin for 5 days, then used for functional studies.

Expression of Serpin A1 in tumour cell lines was analysed by flow cytometry. Briefly, tumour cells were detached using Accutase (Thermo Fisher Scientific, cat# A1105‐01), stained with a viability dye (eBioscience, cat# 65‐0865‐14) in cell suspension, and subsequently fixed and permeabilised using the FoxP3 Fixation/Permeabilisation Kit according to the manufacturer's instructions (BioLegend, cat# 421403). Cells were then incubated with the primary antibody against Serpin A1 (R&D Systems, cat# MAB12681‐SP), followed by staining with the corresponding fluorophore‐conjugated secondary antibodies (BioLegend cat# 405305, 405308).

### 2.5 Cell Lines Supernatants

The cells were cultivated in DMEM (Gibco, cat# 41966‐029) supplemented with 10% of exosome‐depleted (ultracentrifuged at 100,000 × *g* for 18 h) Foetal Bovine Serum (PAN Biotech, cat# P30‐3031), 1% of penicillin‐streptomycin (Gibco, cat# 15140122) and 100 µM non‐essential amino acids (Gibco, cat# 11140050). Cells were grown in a monolayer at 37°C in a humidified incubator with 5% CO_2_. During cultivation, the cell lines were regularly tested for mycoplasma contamination, and the results were negative.

For large production of supernatant enriched with sEVs from the cell lines described above, the 4‐layer Nunc EasyFill Cell Factory System (Thermo Fisher, cat# 140004) was used. Cells were split after 72 h of incubation and re‐seeded using identical cell numbers. Supernatants were harvested before splitting after 72 h of incubation with confluency around 70%–90%.

### 2.6 Isolation of Extracellular Vesicles From Cell Line Supernatants

A 50 mL aliquot of cell culture supernatant was centrifuged at 20°C for 10 min at 2000 × *g* to separate cells and debris. Then supernatants were transferred to new tubes and centrifuged again at 4°C for 30 min at 10,000 × *g*. Afterward, the supernatants were collected and filtered using a 50 mL syringe and a 0.22 µm PES bacterial filter (Millipore, cat# SLGP033RS). In the next step, 50 mL supernatant aliquots were concentrated 50‐fold to 1 mL using Vivacell 100 concentrators with a 100 kDa cut‐off (Sartorius, cat# VS2042) at 3000 × *g*. Small extracellular vesicles were isolated using size‐exclusion chromatography as described for human plasma.

### 2.7 Nanoparticle Tracking Analysis (NTA)

Multiple‐Laser ZetaView f‐NTA Nanoparticle Tracking Analyser (Particle Metrix, Inning am Ammersee, Germany) was used to evaluate the concentration and size distribution of extracellular vesicles. A pre‐testing series (140–200 particles/frame) was performed to assess the proper dilution of sEVs fraction 4. The software settings for sEVs measurement have been selected according to the manufacturer's recommendation. Three repetitions were performed for each sample by scanning 11 cell positions each and capturing 60 frames per position with the following settings: video setting: high; Focus: autofocus; Camera sensitivity for all samples: 92.0; Shutter: 70; Scattering Intensity: 4.0; Cell temperature: 25°C. Captured videos were analysed using the built‐in ZetaView Software with the following parameters: Maximum particle size: 1000; Minimum particle size: 5; Minimum particle brightness: 20; Hardware: embedded laser: 40 mW at 488 nm; camera: CMOS.

### 2.8 Bicinchoninic Acid Assay (BCA)

The protein content was measured using the Pierce BCA Protein Assay Kit (Thermo Scientific, cat# 23225). Prior to measurement, samples were ultrasonicated for 15 min. Next, 10 µL of each sample was added to a 96‐well plate and mixed with 200 µL of working reagent. The plate was incubated for 60 min at 37°C, followed by an absorbance measurement at 560 nm. Protein concentration was determined from a BSA standard curve.

### 2.9 Transmission Electron Microscopy (TEM)

To determine the morphology of sEVs, samples were fixed with 1% osmium tetroxide (OsO_4_) solution (Sigma, cat# 75632–5ML) in a proportion of 1:1 ratio for 30 min. After that, on top of the fixed samples (10 µL), formvar‐coated TEM grids (EMS, cat# EFCF200‐Cu‐TC) were placed for 30 min. In the next step, grids were cleaned out of the fixator by transferring to drops of distilled water (10 µL) for 5 min with three repetitions. Then, grids were placed in drops of 1% uranyl acetate (EMS, cat# 100504‐336) in 50% ethanol (10 µL) for contrast staining for 15 min. In the last step, samples were washed by transferring to drops of distilled water for 5 min with three repetitions. Afterwards, the last drops of water were air‐dried and the grids were ready for TEM. Samples were analysed using a JEM‐1400 Plus transmission electron microscope (JEOL, Peabody, MA, USA), integrated with a TVIPS TemCam‐F416 high‐sensitivity CMOS camera (TVIPS, Martinsried, Germany), which allows taking 16‐bit digital micrographs at a resolution of 4096 × 4096 pixels using the EM‐Menu 4.0 extended software. The contrast, brightness and sharpness of electron micrographs were edited using Adobe Photoshop CC (Adobe, San Jose, CA, USA).

### 2.10 Western Blotting

Ten µL of the SEC fraction—F4 containing 5 µg of protein was mixed with a loading buffer (Bio‐Rad) and optionally 100 mM DTT (in the case when reduction conditions were required), then denatured for 5 min at 95°C. PBS was used as a negative control. A mixture of SEC fractions F5 and F6 derived from healthy volunteers was used as a positive control. Sample separation was performed using 12% SDS‐polyacrylamide gel electrophoresis, followed by wet electrotransfer onto nitrocellulose membranes (Thermo Fisher, cat# 88018). Membranes were blocked for 1 h in 5% non‐fat milk and 0.1% Tween in PBS (for anti‐CD9, anti‐CD63, anti‐TSG101) or in 5% BSA and 0.1% Tween in PBS (for anti‐CD81). Primary antibodies were added for overnight incubation at 4°C (anti‐CD63: Invitrogen, cat# 10628D, 1:1500; anti‐CD9: Santa Cruz, cat# sc‐13118, 1:500; anti‐CD81: Biorbyt, cat# orb388959, 1:500; anti‐TSG101: Becton Dickinson, cat# 612697, 1:800, Serpin A1: R&D Systems, cat# MAB12681‐SP, 1:500, Flotilin‐1: Santa Cruz, cat# sc‐74566). Analysis of CD63 and CD81 on SDS‐PAGE was performed under non‐reducing conditions. The membranes were washed five times with Tris‐buffered saline with Tween (20 mM Tris, pH 7.5, 150 mM NaCl, 0.1% Tween 20) at 20°C, and then the secondary antibody conjugated to HRP, diluted in the same buffer as the primary antibody, was added for 1 h incubation at 20°C. After five washes, Western Bright Sirius HRP substrate (Advansta, cat# K‐12043‐D10) was used for chemiluminescence detection of the bands according to the manufacturer's instructions.

### 2.11 Sample Preparation for LC‐MS/MS

The samples of sEVs (the SEC fraction #4) were thawed and centrifuged: 30 min, 16,000 × *g*, 4°C to remove any suspended matter; then supernatants were collected and subjected to protein precipitation with the use of 10% (m/v) trichloroacetic acid in acetone (precipitant‐to‐sample volume ratio of 3:1, incubation at –20°C for 15 h). Next, samples were centrifuged: 30 min, 16,000 × *g*, 4°C. Supernatants were carefully collected and discarded, and protein pellets were washed twice with 500 µL of pure ice‐cold acetone, each wash followed by centrifugation at 30 min, 16,000 × *g*, 4°C. Leftover acetone was removed by gently heating the tubes in a thermoblock (37°C, ca. 10 min). Each protein pellet was subsequently soaked with 20 µL of 0.2% (m/v) RapiGest in 50 mM NH_4_HCO_3_, mixed, heated to 100°C for 10 min, then cooled to room temperature and spun down briefly. Next, the samples were subjected to protein quantification using the use of tryptophan fluorescence method as published before (Wiśniewski and Gaugaz 2015). Finally, proteins were reduced with dithiothreitol (final concentration of DTT: 5 mM, heating at 60°C for 30 min), alkylated with iodoacetamide (final concentration of IAA: 15 mM, incubation in darkness for 30 min) and subjected to in‐solution digestion using Trypsin/Lys‐C Mix (Promega, cat# V5073) with an enzyme‐to‐protein ratio of 1:50 (m/m). The digestion was carried out at 37°C for 18 h. Next, samples were acidified with trifluoroacetic acid (final concentration of TFA: 0.5%, v/v) and incubated for 45 min at 37°C. Eluates were evaporated to dryness, then reconstituted in water, and the peptide content was determined using the tryptophan fluorescence method; further, the samples were acidified with TFA (final acid concentration: 0.1%). Finally, samples from patients belonging to the same cancer progression stage were pooled. Samples derived from healthy donors (HD) were pooled separately. Peptides present in the pooled samples were preconcentrated using Cleanert C18 1 mL SPE cartridges (Agela Technologies, cat# S180501). Eluates were then evaporated to dryness, reconstituted in water, and peptide content was determined with the use of the tryptophan fluorescence method (Rappsilber et al. 2003). Finally, samples were acidified with TFA (final acid concentration: 0.1%) and frozen until LC‐MS/MS analysis.

### 2.12 Protein Identification and Quantification by LC‐MS/MS

Equal amounts of peptides from the sEV samples were injected into the Dionex UltiMate 3000 RSLC nanoLC System, which was connected to the Q Exactive Plus Orbitrap mass spectrometer (Thermo Fisher Scientific). Next, peptides were separated on a reverse‐phase Acclaim PepMap RSLC nanoViper C18 column (75 µm × 25 cm, 2 µm granulation) using an acetonitrile gradient (from 4% to 60%, in 0.1% formic acid) at 30°C and a flow rate of 300 nL/min (total run time: 180 min). The spectrometer was operated in data‐dependent MS/MS mode with survey scans acquired at a resolution of 70,000 at m/z 200 in MS mode, and 17,500 at m/z 200 in MS2 mode. Spectra were recorded in the scanning range of 300–2000 m/z in the positive ion mode. Higher energy collisional dissociation (HCD) ion fragmentation was performed with normalised collision energies set to 25. A reviewed Swiss–Prot human database was used for protein identification with a precision tolerance of 10 ppm for peptide masses and 0.02 Da for fragment ion masses. A protein was considered positively identified if the search engine found at least two peptides per protein, and if a peptide score reached the significance threshold FDR = 0.01 (as determined by the Percolator algorithm). Furthermore, a protein was classified as ‘present’ if detected in at least one sample of a given type. The abundance of identified proteins was estimated using the Precursor Ions Area detector node in the Proteome Discoverer Software (Thermo Fisher Scientific). This method calculates protein abundance based on the average intensity of each protein's three most intense distinct peptides, normalised to the total ion current (TIC). The mass spectrometry proteomics data have been deposited to the ProteomeXchange Consortium via the PRIDE (Perez‐Riverol et al. 2022) partner repository with the dataset identifier PXD057119.

### 2.13 Evaluation of Serpin A1 Cargo Localisation Using Western Blotting

Small EVs (SEC fraction F4) in PBS, at a final concentration of 200 µg/mL were treated with proteinase K (Thermo Fisher Scientific, cat# EO0491; final concentration 50 µg/mL) and Triton X‐100 (Sigma–Aldrich, cat# T9284‐100ML; final concentration 0.1%) for 1 h at +37°C. The reaction was stopped by addition of phenylmethanesulfonylfluoride (PMSF, Sigma–Aldrich, cat# P7626‐250MG; final concentration 2 mM) for 5 min. The sEV samples were denatured at 65°C for 10 min. Firstly, 30 µg total sEV was subjected to 10% SDS‐PAGE gel electrophoresis and transferred to nitrocellulose membranes (Thermo Fisher, cat# 88018). Subsequently, the membrane was blocked for 1 h in 5% non‐fatty milk and 0.1% Tween in TBS, then incubated with the primary antibody against Serpin A1 (R&D Systems, cat# MAB12681‐SP) overnight at 4°C. Next, after washing with TBST buffer, the membranes were incubated with the appropriate HRP‐conjugated antibody for 1.5 h at room temperature. After five washes, Western Bright Sirius HRP substrate (Advansta, cat# K‐12043‐D10) was used for chemiluminescence detection of the bands according to the manufacturer's instructions.

### 2.14 Preparation of EVs‐Depleted Cell Culture Medium

Cell culture medium was prepared according to the protocol of Théry et al. (2006). Briefly, the medium complemented with all required nutrients and 20% (v/v) FBS, was ultracentrifuged for 18 h at 100,000 × *g* at 4°C (45Ti rotor, Beckman Coulter, cat# 339160). Then, the collected supernatant was sterilised using a 0.22‐µm syringe filter and stored at 4°C. The desired concentration was obtained by diluting the complete medium without FBS.

### 2.15 Isolation of Human Neutrophils and T Cells

Peripheral blood was drawn into 3.8% sodium citrate anticoagulant monovettes (Sarstedt, cat# 02.1067.001) and mixed 1:1 with PBS (Gibco, cat# 14040133) before separation by density gradient centrifugation (Biocoll density 1077 g/mL, Merck, cat# L6115) for 30 min at 300 × *g* (without a break) at 20°C. The mononuclear cell fraction was collected, resuspended in buffer (PBS, 0.5% bovine serum albumin (R&D, cat# 5217), 2 mM EDTA (Sigma–Aldrich, cat# E9884)) and centrifuged at 300 × *g* 4°C (repeated three times to minimise contamination with platelets). T cells were isolated from mononuclear cells using immunomagnetic separation (pan T cell negative selection kit, Miltenyi, cat# 130‐096‐535) according to the manufacturer's protocol. Isolated T cells (purity ∼95%) were stored on ice in RPMIc medium (RPMI 1640 Medium (Gibco, cat# 11875093), 10% of exosome‐depleted (ultracentrifuged at 100,000 × *g* for 18 h) Foetal Bovine Serum (PAN Biotech, cat# P30‐3031) and 1% penicillin‐streptomycin (Gibco, cat# 15140122)) for 1 h. Neutrophils were isolated from the polymorphonuclear cell fraction by sedimentation over 1% polyvinyl alcohol (Sigma–Aldrich, cat# 360627) for 30 min at 20°C, followed by hypotonic lysis of erythrocytes with 0.2% NaCl solution for 1 min at 20°C and reconstitution of osmolarity with 1.2% NaCl. Isolated neutrophils (purity ∼95%, Figure S1) were resuspended in RPMIc medium at 20°C to reach a concentration of 4 × 10^6^ cells/mL.

### 2.16 Internalisation of sEVs by Neutrophils

The ability of tumour‐derived sEVs to be internalised by neutrophils was investigated by examining the uptake of vesicles isolated using SEC and labelled with the CFSE dye (BioLegend, cat# 423801). To perform CFSE staining, 15 µL of a 40 µM CFSE solution was added to 100 µL of PBS, containing 10 µg of sEVs. This mixture was then incubated for 45 min at +37°C, 5% CO_2_. To eliminate unbound CFSE dye, we used size‐exclusion chromatography as described previously. Concurrently, freshly isolated neutrophils were co‐incubated together with DID (Biotium, cat# 60014–5 mg) to label the cell surface and PureBlu Hoechst 33342 (BioRad, cat# 1351304EDU) to visualise the DNA, following the manufacturers' instructions for both stains. Next, 5 × 10^5^ of neutrophils were applied to a glass‐bottom 96‐well plate (MatTek, cat# P96G‐1.5‐5‐F) precoated with poly‐D‐lysine 1 mg/mL (Sigma–Aldrich, cat# P0899‐10MG) for 4 h at +37°C, 5% CO_2_. The plates were then centrifuged for 5 min at 300 × *g* to ensure cell adhesion. After removing the supernatant, 100 µL of PBS containing 10 µg of sEVs were added to the wells, followed by a 2‐h incubation at +37°C, 5% CO_2_. The plates were washed twice with 200 µL of PBS and fixed with 4% paraformaldehyde (Alfa Aesar, cat# 43368). Images were collected on Zeiss LSM 710 combined with ELYRA PS.1 Super‐resolution and TIRF (total internal reflection fluorescence) module using 100×, 1.46 NA Zeiss objective and GaASP detector (Carl Zeiss, Germany). Cell mid‐sections were acquired at 12‐bit image depth with line averaging (setting 4) and XY pixel size 83 nm in the following channels: DID (Ex: 646 nm; Em: 663 nm), CFSE (Ex: 488 nm; Em: 490–553 nm), PureBlue Hoechst (Ex: 350 nm; Em: 461 nm). Images were scaled to 8‐bit RGB in Zen software (Carl Zeiss) and exported as JPEGs.

### 2.17 Inhibition of sEV Internalisation

To determine whether active vesicle internalisation is required for sEV‐induced neutrophil reprogramming, freshly isolated neutrophils were pre‐treated for 1 h at 37°C with either Genistein (Abcam, cat# AB120112; final concentration 100 µM), an inhibitor of clathrin/caveolin‐mediated endocytosis, or CK‐666 (Adooq, cat# A20973‐1; final concentration 100 µM), an inhibitor of Arp2/3‐dependent actin branching required for macropinocytosis, prior to co‐incubation with td‐sEVs (final concentration 50 µg protein/mL) or PBS for 18 h at 37°C, 5% CO_2_. Cells were then analysed by flow cytometry for viability, CD11b, PD‐L1, STAT3 and pSTAT3 as described in the flow cytometry section.

### 2.18 Education of Neutrophils Using EVs

To investigate changes in neutrophil phenotype and functions under the influence of tumour‐derived sEVs, neutrophils (5 × 10^5^ in 200 µL of RPMIc) were co‐incubated with 10 µg of unlabelled tumour cell‐derived sEVs in 50 µL PBS (or PBS alone as a negative control) in 24‐well flat‐bottom plates for 1 h (further co‐incubation with tumour cells and T cells, see below) or 18 h (for evaluation of survival and phenotypical changes, see below) and cultured at 37°C as described above. To diminish the effects of td‐sEVs, an inhibitor of STAT3 phosphorylation, LLL12, in a concentration of 2.5 nM/mL (Sigma–Aldrich, cat# 573131), was added together with td‐sEVs. To evaluate the contribution of serpin‐primed Arginase 1 production in neutrophils on their immunosuppressive potential, an inhibitor of Arginase 1, Numidargistat (Synonyms: CB‐1158; INCB01158) (MedChemExpress, cat# HY‐101979), at a concentration of 200 nM, was added after neutrophil priming together with T cells.

### 2.19 Survival Assay

Spontaneous apoptosis of neutrophils isolated from human blood was determined by Annexin V/7‐aminoactinomycin (7AAD) apoptosis detection kit (BD, cat# 559763) after 18 h of incubation in RPMIc medium, with or without sEVs. The percentages of alive (annexin V^–^/7‐AAD^–^), apoptotic (early apoptosis, annexin V^+^/7‐AAD^–^) and dead by apoptosis (late apoptosis, annexin V^+^/7‐AAD^+^) cells from all single cells were estimated.

### 2.20 Flow Cytometry

Single‐cell suspensions were stained with eBioscience Fixable Viability Dye (eBioscience, cat# 65‐0865‐14) and specific antibodies (see Resources table). Staining was performed for 30 min at 4°C.

For analysis of intracellular Arginase 1 in neutrophils sEVs‐priming, monensin (BioLegend, cat# 420701) and brefeldin (BioLegend, cat# 420601) were added after 2 h of sEVs stimulation to prevent the release of produced Arginase 1 for 18 h, cells then were stained with a viability dye (eBioscience, cat# 65‐0865‐14) in cell suspension, and subsequently fixed and permeabilised using the FoxP3 Fixation/Permeabilisation Kit according to the manufacturer's instructions (BioLegend, cat# 421403). Neutrophils were then incubated with antibodies against Arginase 1 (BioLegend, cat# 369706).

For analysis of STAT3 and pSTAT3 expression in neutrophils after 18 h EV‐priming, cells were stained with a viability dye (eBioscience, cat# 65‐0865‐14) in cell suspension, then fixed with 96% methanol at –20°C within 1 h, with subsequent fixation and permeabilisation using the FoxP3 Fixation/Permeabilisation Kit (BioLegend, cat# 421403). Neutrophils were then incubated with antibodies against STAT3 (Invitrogen, cat# MA5‐23635) and pSTAT3 (y705) (BioLegend, cat# 651004) and analysed with flow cytometry.

### 2.21 Multiparameter Flow Cytometry

Neutrophils (4 × 10^6^ cells/ mL) were incubated with PBS, WT or A1‐KO td‐sEVs (each group receiving an equal sEVs protein load standardised by BCA assay) for 18 h in 24‐well plates at 37°C, 5% CO_2_. For downstream immunophenotyping, cells were first stained with fluorochrome‐conjugated antibodies for surface antigens (including CD11b, CD66b, MHC‐I, PD‐L1 and CD62L). After primary surface staining, cells were fixed with 96% methanol for 1 h at −20°C to preserve cell integrity and antigenicity. Subsequently, samples were permeabilised using the FoxP3 Fixation/Permeabilisation kit (BioLegend, cat# 421403) and stained for intracellular antigens, including phosphorylated STAT3 (pSTAT3), total STAT3, Arginase 1 and IL‐10.

Flow cytometric data were acquired on a BD Symphony A3 cytometer. All events were concatenated in FlowJo v10.8.1 using the concatenation tool, ensuring equal representation of each experimental condition for unbiased computational analysis. Dimensionality reduction and cluster identification were employed using t‐Distributed Stochastic Neighbour Embedding (tSNE, opt‐SNE implementation) and X‐shift density‐based clustering algorithms within FlowJo's Cytobank plug‐in. Marker expression was visualised and quantified across clusters, with cellular subsets manually annotated based on canonical neutrophil surface and functional markers.

### 2.22 Proteinase K Protection Assay

To assess the accessibility of Serpin A1 on the surface of td‐sEVs, sEV preparations (SEC fraction F4) were incubated with Proteinase K (Thermo Fisher, cat# EO0491; final concentration 50 µg/mL) for 1 h at 37°C. The reaction was stopped by addition of Phenylmethansulfonylfluorid (PMSF, Sigma–Aldrich, cat# P7626‐250MG; final concentration 2 mM) for 5 min. Proteinase K‐treated or vehicle‐treated (PBS) sEVs were subsequently applied to freshly isolated neutrophils (final concentration 50 µg protein/mL) for 18 h at 37°C, 5% CO_2_, followed by flow cytometric analysis of viability, CD11b, PD‐L1, STAT3 and pSTAT3 as described above.

### 2.23 CD91/LRP1 Receptor Blocking Assay

To evaluate the contribution of CD91/LRP1‐mediated engagement of Serpin A1 to neutrophil reprogramming, freshly isolated neutrophils were pre‐incubated with a blocking anti‐CD91/LRP1 antibody (BioXCell, cat# be0333; final concentration 75 µg/mL) for 1 h at 37°C, prior to co‐incubation with td‐sEVs (final concentration 50 µg protein/mL) or PBS for 18 h at 37°C, 5% CO_2_. Cells were subsequently analysed by flow cytometry for viability, CD11b, PD‐L1, STAT3 and pSTAT3 as described above.

### 2.24 ROS

Spontaneous and PMA‐induced ROS production in neutrophils incubated with PBS, WT (Serpin A1‐sufficient), or A1‐KO (Serpin A1‐deficient) td‐sEVs (final concentration 50 µg protein/mL) for 18 h was measured using Dihydrorhodamine 123 (Sigma–Aldrich, cat. D1054‐2MG) according to the manufacturer's instructions. Where indicated, cells were stimulated with phorbol 12‐myristate 13‐acetate (PMA; Biomol, cat# Cay‐10008014‐1; final concentration 20 nM) for 30 min at 37°C prior to measurement. Fluorescence intensity was assessed by flow cytometry as described above.

### 2.25 NET Formation Assay

Spontaneous and PMA‐induced NET formation in neutrophils incubated with PBS, WT or A1‐KO td‐sEVs (final concentration 50 µg protein/mL) for 18 h was assessed using SYTOX Green (Thermo Fisher, cat# S7020) as a marker of extracellular DNA release, according to the manufacturer's instructions. Where indicated, cells were stimulated with PMA (final concentration 20 nM) for 30 min at 37°C prior to measurement. Fluorescence intensity was measured by flow cytometry as described above.

### 2.26 Treatment of Neutrophils With Soluble Recombinant Serpin A1

To assess whether soluble, vesicle‐free Serpin A1 is sufficient to reproduce the effects of td‐sEVs, freshly isolated neutrophils (4 × 10^6^ cells/mL) were incubated with recombinant human Serpin A1 protein (Novus Biologicals, cat# 1268‐PI‐010) at a final concentration of 1 µg/mL in RPMIc medium, or with PBS as a negative control, for 18 h at 37°C, 5% CO_2_. After incubation, cells were processed for flow cytometric analysis of viability, CD11b, PD‐L1, STAT3, pSTAT3 and Arginase 1, as described above.

### 2.27 Induction of EMT by Neutrophils Primed by Tumour‐Derived sEVs

Isolated neutrophils were co‐incubated with td‐sEVs or PBS as a negative control (as described above) for 1 h. Td‐sEVs not internalised by neutrophils, were removed by washing using PBS and centrifugation at 300 × *g* for 5 min, 20°C, repeated three times. Primed neutrophils were mixed with UT‐SCC‐50 tumour cells in the ratio 10:1 (150,000 neutrophils to 15,000 tumour cells) in 200 µL of DMEMc in a 48‐well plate in duplicate for each biological replicate and incubated for 24 h at 37°C, 5% CO_2_. After incubation, the wells were washed gently with PBS to remove neutrophils and dead tumour cells. The UT‐SCC‐50 cell line was preferred due to its early stage and more prominent epithelial phenotype, which is suitable for investigating epithelial‐to‐mesenchymal transition (EMT).

To evaluate the morphological changes of the tumour cells driven by td‐sEVs‐primed neutrophils, well‐alive adherent tumour cells remaining in the culture were fixed with 1 mL of ice‐cold 96% methanol for 1 h at –20°C, followed by staining with 0.01% aqueous solution of gentianine (Santa Cruz, cat# CAS 439‐89‐4) for 10 min. The plate was washed with dH_2_O and left to dry overnight. The amount and morphology of tumour cells after co‐incubation with td‐sEVs‐primed neutrophils were evaluated using AMG EVOS digital phase‐contrast inverted microscope (in 4–8 fields of view, four biological replicates) and analysed using FIJI (ImageJ) software (https://fiji.sc/).

To evaluate the EMT gene reprogramming of tumour cells induced by td‐sEVs‐primed neutrophils, the remaining adherent tumour cells in the well were stored in 50 µL of RNA*later* solution (Invitrogen, cat# AM7020) at –20°C. The RNA was isolated using QIAShredder (Qiagen, cat# 79656) and RNeasy Mini Kit (Qiagen, cat# 74106), and the cDNA was produced using the Superscript II Reverse Transcriptase Kit (Invitrogen, cat# 18064014). qRT‐PCR was performed at 60°C annealing temperature using the following primers:

VIM_f AGGCAAAGCAGGAGTCCACTGA, VIM_r ATCTGGCGTTCCAGGGACTCAT,

TJP1_f GTCCAGAATCTCGGAAAAGTGCC, TJP1_r CTTTCAGCGCACCATACCAACC,

ARG1_f TCATCTGGGTGGATGCTCACAC, ARG1_r GAGAATCCTGGCACATCGGGAA,

SNAIL_f TGCCCTCAAGATGCACATCCGA, SNAIL_r GGGACAGGAGAAGGGCTTCTC,

SLUG_f ATCTGCGGCAAGGCGTTTTCCA, SLUG_r GAGCCCTCAGATTTGACCTGTC,

TWIST_f GCCAGGTACATCGACTTCCTCT, TWIST_r TCCATCCTCCAGACCGAGAAGG,

ZEB1_f GGCATACACCTACTCAACTACGG, ZEB1_r TGGGCGGTGTAGAATCAGAGTC,

ZEB2_f AATGCACAGAGTGTGGCAAGGC, ZEB2_r CTGCTGATGTGCGAACTGTAGG.

Additionally, *ACTB* housekeeping gene was used as a reference: ACTB_f CACCATTGGCAATGAGCGGTTC, ACTB_r AGGTCTTTGCGGATGTCCACGT. The mRNA expression was measured using the Luna Universal qPCR Master Mix (BioLabs, cat# M3003X). Relative gene expressions were calculated by 2^–ΔCt formulations in biological replicates *n* = 4–6.

### 2.28 Induction of T Cell Exhaustion by Td‐sEVs‐Primed Neutrophils

Neutrophils were co‐incubated with td‐sEVs or PBS as a negative control for 1 h, followed by washing with PBS (centrifugation at 300 × *g* for 5 min, 20°C, repeated three times) to remove td‐sEVs that were not internalised by neutrophils. Primed neutrophils were co‐incubated with autologous T cells at a ratio of 1:1 (150,000 neutrophils to 150,000 T cells) in 200 µL of RPMIc containing aCD3 (BioLegend, cat# 300402), aCD28 (BioLegend, cat# 302902) and rhIL‐2 (Peprotech, cat# 200–02) in a 48‐well plate and incubated for 24 h at 37°C 5% CO_2._ After priming of T cells by neutrophils, UT‐SCC‐50 tumour cells were added in a proportion of 1:10 (15,000 tumour cells in 50 µL of DMEMc) to the wells and further incubated for 24 h at 37°C 5% CO_2._ Then, the supernatant and non‐adherent neutrophils and T cells were collected, and the exhaustion state of T cells (alive CD4^+^ and CD8^+^) was estimated based on the expression of PD‐1 (BioLegend, cat# 367412) using flow cytometry. The killing capacity of stimulated T cells was assumed to be negatively proportional to the density of the remaining alive adherent UT‐SCC‐50 tumour cells and was evaluated with AMG EVOS digital phase contrast inverted microscope (in 4–8 fields of view, four biological replicates) and analysed by FIJI (ImageJ) software (version 2.9.0).

### 2.29 Analysis of the Data Deposited in the Gene Expression Omnibus Databases

We used previously published microarray data deposited in the Gene Expression Omnibus database, GSE83519 and GSE65858 (Wichmann et al. 2015) (Table S1).

### 2.30 Bioinformatical and Statistical Analysis

The Perseus software (version 1.6.14) was used to perform statistical and bioinformatic analysis of mass spectrometry‐based proteomic data. To explore differences between the analysed groups, we used only proteins identified in at least 80% of the samples in each group. The imputation of missing values was made based on the default parameters of the Perseus software. To determine differences in protein expression, LFQ intensity (LFQI) was used as an indicator of each protein's abundance across samples. The significance of differences in protein abundance was estimated using an ANOVA Multiple‐sample Test of the log2‐transformed LFQI values followed by Benjamini–Hochberg correction. In addition, to estimate the magnitude of such differences, we calculated fold changes as the ratios of the median LFQI values for each protein in the compared groups. Functional Enrichment analysis was performed based on Cytoscape (Shannon et al. 2003) (https://cytoscape.org/) open source software platform using EnrichmentMap (Merico et al. 2010) (https://apps.cytoscape.org/apps/enrichmentmap) and AutoAnnotate (https://apps.cytoscape.org/apps/autoannotate) applications. Hierarchical clustering was performed in Morpheus (https://software.broadinstitute.org/morpheus) to visualise differences in expression patterns. ShinyGO was used to visualise overrepresented pathways (Ge et al. 2020) (http://bioinformatics.sdstate.edu/go/). Volcano plots were generated using VolcaNoseR (Goedhart and Luijsterburg 2020) (https://huygens.science.uva.nl/VolcaNoseR2/). Statistical analyses were performed using Kruskal–Wallis ANOVA for multiple comparisons with the Bonferroni correction, the Mann‐Whitney *U*‐test for two independent samples, and the Wilcoxon test for dependent samples. The correlations between variables were analysed using the Spearman R test (visualised at https://www.bioinformatics.com.cn/ [Tang et al. 2023]). Kaplan–Meier curves for the survival function were compared via log‐rank test. Data are shown as median with interquartile range or as individual values with median. *p* < 0.05 was considered significant.

## 3 Results

### 3.1 Elevated Content of sEVs in Plasma During HNC Progression

To analyse the impact of cancer on the plasma small extracellular vesicle (sEV) compartment, we compared count and characteristics of sEVs between HNC patients and healthy individuals. sEVs were isolated from the plasma of patients at different stages of cancer disease as well as from healthy donors (HD) (Figure 1A,B; **Table** S2 and Figure S2) as described previously (Hong et al. 2016, 2017; Ludwig et al. 2019). The quality of isolated sEVs was evaluated in agreement with MISEV2023 (Welsh et al. 2024) guidelines. The presence of the common sEVs markers (CD9, CD63, CD81, TSG101 and Flotillin‐1) was confirmed by western blotting (Figure 1C). Morphologies of plasma‐derived sEVs were analysed using transmission electron microscopy (TEM) (Figure 1D). Particle concentration and sizes within obtained plasma‐derived sEV samples were determined by nanoparticle tracking analysis (NTA) (Figure 1E‐G). No differences were observed regarding sEV morphology or the average particle sizes among sEV plasma samples of healthy donors (HD) and patients with HNC at different stages; in all samples particle sizes were in the range between 50 and 150 nm (Figure 1D‐F). However, significant higher particle quantities were recovered in sEV samples from plasma of HNC patients than in those from healthy donors (Figure 1G). Of note, the protein content in plasma‐derived sEVs isolated from patients with an advanced stage of disease (UICC stage III/IV) was significantly higher as compared to those of an early cancer stage (UICC stage I/II) or HD: HD = 44 µg/mL, UICC I/II = 57.3 µg/mL, UICC III/IV 75.7 µg/mL (Figure 1H).

These findings imply a stage‐dependent increase in sEVs abundance in HNC patients’ plasma, establishing a foundation for exploring the mechanistic impact of sEV‐associated proteins in cancer‐related immune modulation.

### 3.2 Cancer Associated sEVs Are Enriched in Serpins

To identify tumour‐associated cargo of plasma‐derived sEVs (pd‐sEVs), we investigated changes in the proteome composition of sEV samples from HNC versus healthy donors using LC‐MS/MS with label‐free quantification (LFQ). Individual samples from the same group (HD, HNC‐I, HNC‐II, HNC‐III and HNC‐IV) were pooled, and MS‐based analysis was performed in triplicate for each pooled sample (Table S3). Among differentially expressed proteins (DEPs) upregulated in sEVs from HNC patients, with either low (UICC I/II) or high stage (UICC III/IV), there were members of the serpin family, such as Serpin A1, Serpin A3 or Serpin G1, which showed stage‐related abundance (Figure 2A,B). Analysis of enrichment pathways reflected by the proteome of the HNC‐associated sEV samples revealed pathways potentially involved in the modulation of neutrophil activity, such as metabolic process regulation, apoptotic signalling, kinase phosphorylation and neutrophil‐mediated immunity (Figure S3). Serpins were key components of the interactome formed by sEVs proteins highly abundant in HNC patients (Figure 2B). Noteworthy, serpins are a family of proteins with inhibitory activity towards neutrophil granule proteases, while dysregulated neutrophil degranulation plays an important role in HNC progression (Lecot et al. 2022).

**FIGURE 2:**
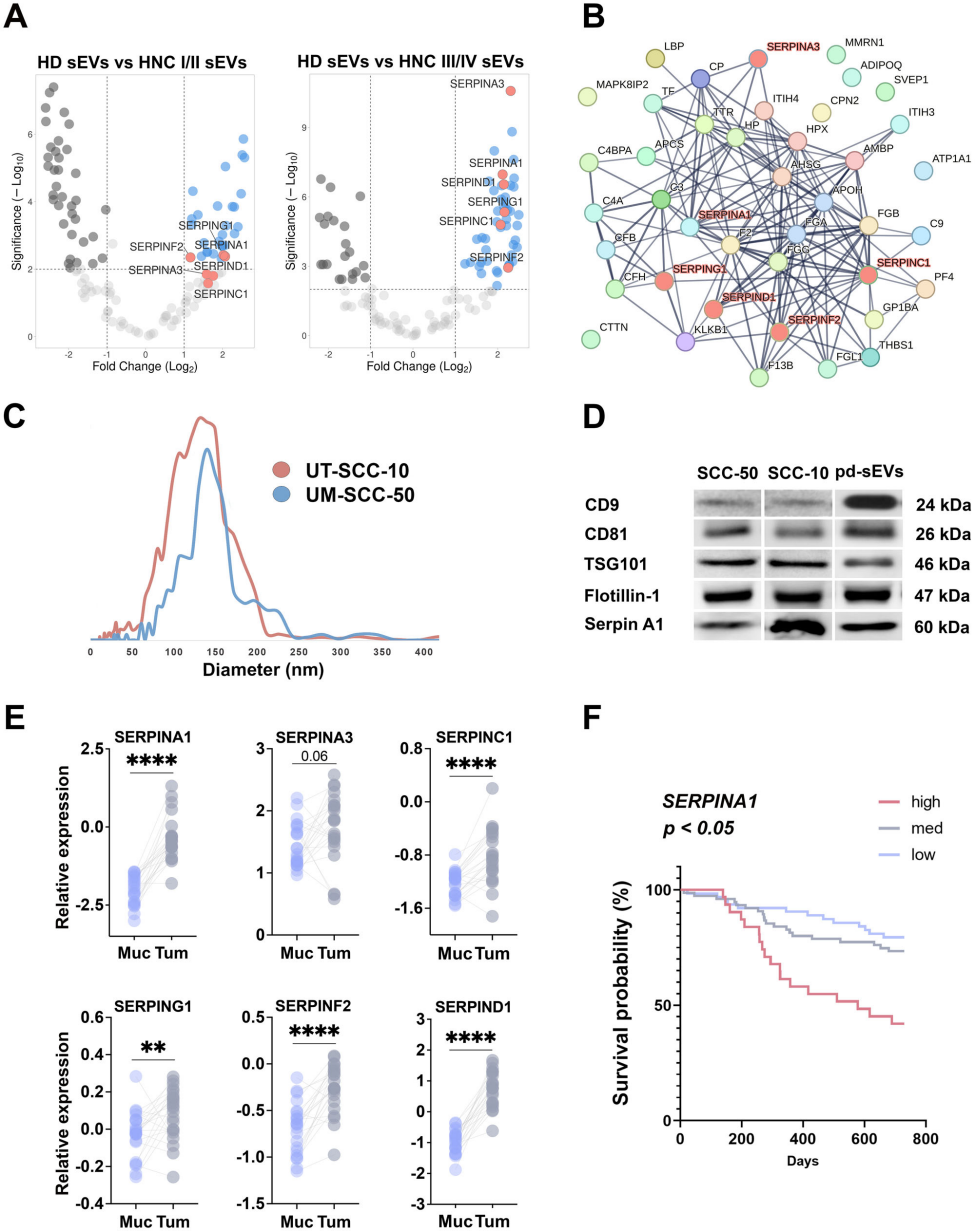
Serpin upregulation parallels cancer progression. (A) Volcano plots illustrating fold change (log2, x‐axis) versus FDR‐adjusted *p* value (significance, −log10, y‐axis) of proteins within plasma‐derived sEVs samples. Data are represented as results obtained for the control group—healthy donors relative to UICC I∖II and UICC III∖IV. Changes were considered significant at *p* < 0.01 and fold change > –1.5 (downregulation in grey) or <1.5 (upregulation in blue) compared with the control group. The grey perpendicular puncture line indicates the border between proteins that have passed a significant value. (B) STRING‐based enrichment analysis of proteins that are upregulated in pd‐sEVs from HNC patients compared to those from healthy donors. Among others, we could identify several members of the serpin family (Serpin A1, A3, C1, G1, D1, F2), thereby validating their association with the tumour. (C) Nanoparticle tracking analysis (NTA) of sEVs isolated from supernatants of tumour HNC‐derived cell lines UT‐SCC‐10a (pink) and UM‐SCC50 (blue) revealed similar mean sizes. (D) Western blotting confirmed the presence of common markers such as CD9, CD81, CD63, TSG101, Flotillin‐1 and Serpin A1 in sEVs derived from UM‐SCC50 (SCC‐50) and UT‐SCC‐10a (SCC‐10) and plasma (pd‐sEVs). (E) Analysis of the open‐access database GSE83519 shows relative expression levels of various serpin proteins (Serpin A1, A3, C1, D1, F2 and G1) in tumour tissue (Tum) compared to healthy mucosa (Muc). (F) Analysis of the open‐access database GSE6585827 demonstrates an association of Serpin A1 overexpression in tumour tissue as a predictor of poor prognosis in HPV‐negative HNC. For the comparison of two independent groups, the Mann–Whitney U test was used. For the comparison of two dependent groups, the Wilcoxon test was used; comparison of Kaplan–Meier survival curves by Log‐rank (Mantel–Cox) test. *p < 0.05, **p < 0.01, ***p < 0.005, ****p < 0.0001.

Plasma contains a heterogeneous mixture of EVs derived from various cell types. Therefore, to provide evidence for the presence of serpins specifically in tumour‐derived EVs, we purified sEVs from HNC cell lines derived from either early stage (T2N0) or the advanced stage (T3N0M0) tumours: UT‐SCC‐50 and UM‐SCC‐10a, respectively. Small EVs released by both types of HNC cell lines in vitro showed the same size (approx. 130 nm according to NTA) (Figure 2C; Figure S4). Western blot analysis of such td‐sEVs confirmed the presence of Serpin A1 in their cargo, as well as of the *bona fide* sEVs markers (CD9, CD81, TSG101, Flotillin‐1) (Figure 2D). To assess the purity of our sEVs preparations and exclude contamination with endoplasmic reticulum derived membrane structures, Western blot analysis for Calnexin was performed (Figure S5).

To evaluate the role of serpins in the development of HNC, we used publicly available transcriptomic datasets (GSE83519, GSE65858 [Wichmann et al. 2015]) and compared the expression levels of serpin‐encoding genes in HNC tumour tissue and corresponding healthy mucosa. This analysis revealed an upregulation of serpin transcripts in tumour tissue (Figure 2E). Moreover, an overexpression of serpins in tumour tissue was associated with disease progression and poor prognosis in HNC (Figure 2F).

### 3.3 Tumour‐Derived sEVs Promote Survival and Pro‐Tumoural Phenotype of Neutrophils

To evaluate the effect of td‐sEVs on neutrophil activity, we applied aliquots of CFSE labelled sEV samples to naïve neutrophils and observed efficient internalisation of sEVs (Figure 3A), followed by significant changes in neutrophil proteome and functionality (Figure 3B‐F). We observed significant alterations of proteins involved in neutrophil activation and degranulation processes, regulation of programmed cell death, as well as regulation of adaptive immune responses, such as antigen processing and presentation. Furthermore, neutrophils treated by sEVs from early‐stage (UT‐SCC‐50) or late‐stage (UM‐SCC‐10a) cancer exhibited pathway enrichment profiles (Figure 3C) that were comparable to those found in neutrophils isolated from early‐ or late‐stage HNC patients (Figure 3D; Table S4).

**FIGURE 3:**
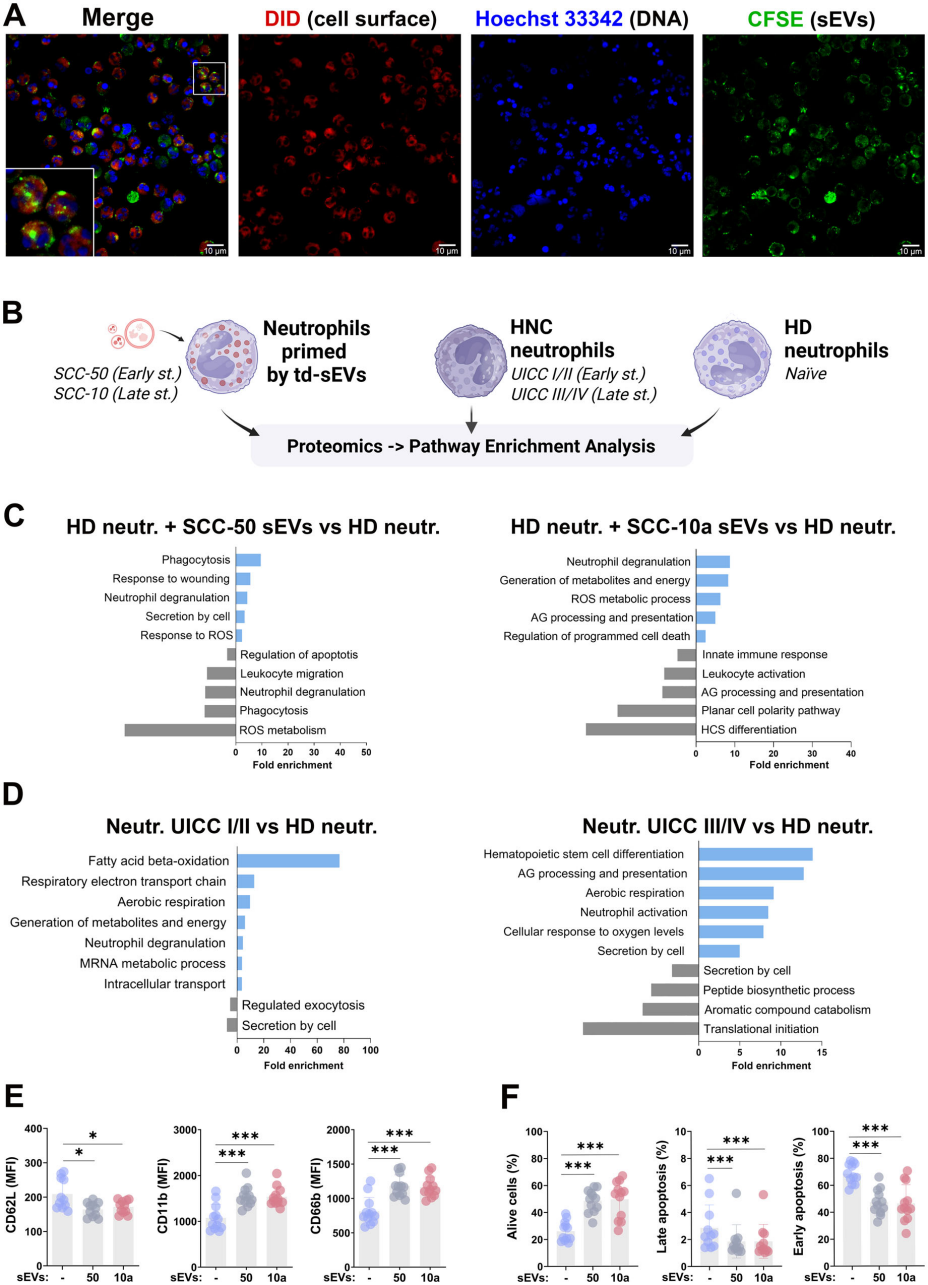
Treatment with tumour‐derived sEV samples triggers activation of neutrophils. (A) Uptake experiments were performed with sEV samples after CFSE labelling and purification via size‐exclusion chromatography (SEC). Neutrophils were co‐incubated together with DID to label the cell surface and PureBlu Hoechst 33342 to visualise the DNA. Scale bar 10 µm. (B) Scheme of experimental model: the proteome profile of neutrophils primed by td‐sEVs from the UT‐SCC‐50/UM‐SCC‐10a cells was compared to proteome of neutrophils isolated from head and neck (HNC) patients or healthy donors (HD). (C) Overrepresented processes associated with proteins upregulated in HD‐derived neutrophiles incubated with td‐sEVs versus untreated controls. (D) Overrepresented processes associated with proteins upregulated in neutrophils isolated from the plasma of HNC patients versus untreated controls. (E) Freshly isolated human neutrophils treated in vitro with td‐sEVS showed elevated cell activation, characterised by downregulation of CD62L and increased expression of CD11b and CD66b. Expression of CD62L, CD11b and CD66b on neutrophils was estimated by flow cytometry. (F) The percentages of alive (annexin V–/7‐AAD–), apoptotic (annexin V+ /7‐AAD‐) and dead by apoptosis (annexin V+/7‐AAD+) neutrophils after education with td‐sEVs were estimated by flow cytometry, control (control marked as ‘‐’, HD neutrophils), 50 (HD neutrophils treated by UM‐SCC‐50 sEVs), 10a (HD neutrophils treated by UT‐SCC‐10a sEVs). Representative results from three replicate experiments are shown. For the comparison of two dependent groups, the Wilcoxon test was used. Data are shown as individual values with a median. *p < 0.05, ***p < 0.005.

These findings reveal that activation of neutrophils in vitro by td‐sEVs mirrors the proteomic alterations observed in neutrophils from HNC patients.

In line with the proteomics data, we observed elevated neutrophil activation and degranulation after education with td‐sEVs, as shown by the downregulation of CD62L and increased expression of CD11b and CD66b (Figure 3E). Next, we assessed the lifespan of such neutrophils and measured percentages of alive (annexin V^–^/7‐AAD^–^), apoptotic (early apoptosis, annexin V^+^ /7‐AAD^–^) or dead by apoptosis (late apoptosis, annexin V^+^/7‐AAD^+^) cells (Figure 3F). In agreement with previously observed prolonged lifespan of pro‐tumoural neutrophils in cancer patients, education with td‐sEVs significantly increased the lifespan of these cells and reduced their apoptosis (Figures 3F, S6).

To exclude the possibility that soluble tumour‐derived factors, rather than td‐sEVs, are responsible for the observed phenomenon, neutrophils were treated in parallel with sEV‐depleted, tumour‐derived supernatants. Importantly, no alterations of their phenotype were detected (Figure S7). The obtained results demonstrate that the changes of neutrophil phenotype and activity during cancer progression are attributed to td‐sEVs, rather than soluble factors released by cancer cells to plasma or tumour microenvironment.

### 3.4 Tumour‐Derived sEV‐Educated Neutrophils Induce EMT in Cancer Cells via Serpin A1

To functionally assess properties of sEV‐reprogrammed neutrophils, we incubated sEV‐primed neutrophils with cancer cells (Figure 4A). We observed that pd‐sEVs from cancer patients, did not stimulate neutrophil cytotoxicity towards cancer cells, while pd‐sEVs from healthy volunteers elicited strong cytotoxic activity of these cells (Figure 4B). Considering the presence of serpins in sEVs released by cancer cells, we aimed to evaluate the importance of those proteins in the reprogramming of neutrophils.

**FIGURE 4:**
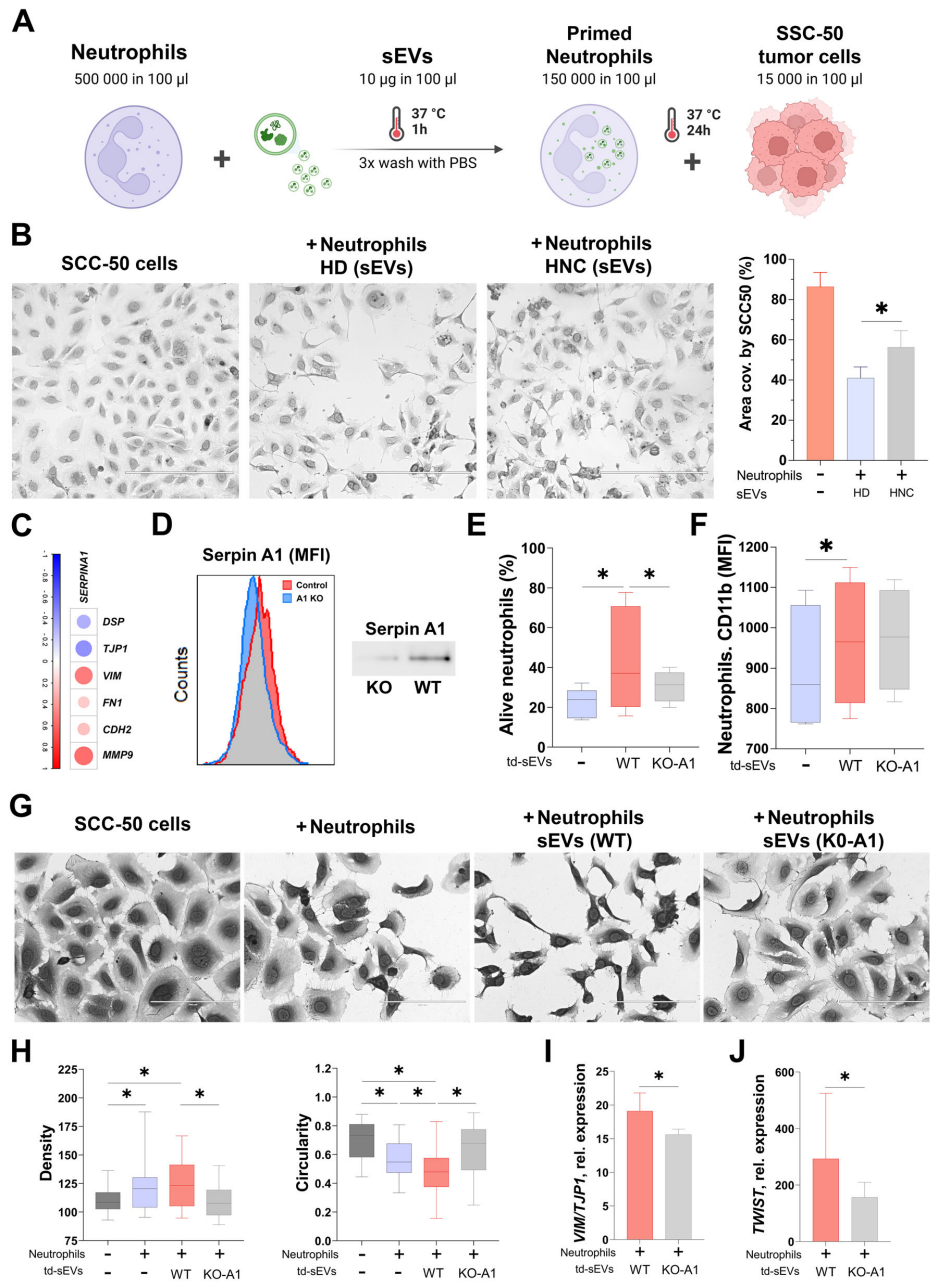
Tumour‐derived serpin^hi^ sEVs induce EMT through neutrophil reprogramming. (A) Scheme of experiment model: neutrophils primed by sEVs from the UM‐SCC‐10a cells were co‐cultivated with UT‐SCC‐50 cells. (B) The viability of UT‐SCC‐50 cells co‐incubated with neutrophils primed by plasma‐derived sEVs purified from the blood of HD and HNC patients (cancer cells cultured in the absence of neutrophils are the reference), scale bar 400 µm; the graph on the right represents quantitatively the area covered by cancer cells. (C) The correlation between the expression of *SERPINA1* and the expression of selected genes related to the EMT in HNC tumour tissue (analysis was based on the dataset GSE65858^27^). (D) The presence of Serpin A1 was analysed by flow cytometry and Western blotting in UM‐SCC‐10a cells after the CRISPR/Cas knockout (KO) of the corresponding gene. Viability (E) and expression of CD11b (F) in neutrophils incubated with td‐sEVs from the original serpin^hi^ UM‐SCC‐10a cells (WT) and serpin^def^ Serpin A1 (compared to control neutrophils not incubated with td‐sEVs). (G) Morphology of UT‐SCC‐50 cells co‐cultivated with td‐sEVs‐primed neutrophils. Cancer cells cultured in the absence of neutrophils and co‐cultured with untreated neutrophils are shown as references (representative images are shown; scale bar 200 µm). (H) The analysis revealed significant differences in cell density and circularity, indicating mesenchymal transformation. (I) The proportion of *VIM* and *TJP1* gene transcripts in UT‐SCC‐50 cells co‐cultivated with td‐sEVs‐primed neutrophils from either WT or KO‐A1 UM‐SCC‐10a cells. (J) *TWIST* mRNA expression in UT‐SCC‐50 tumour cells co‐cultivated with td‐sEVs‐primed neutrophils from either WT or KO‐A1 UM‐SCC‐10a cells. For the comparison of two dependent groups, the Wilcoxon test was used. Data are shown as bars with the median indicated. Correlations were analysed with the Spearman *R* test. *p < 0.05.

First, we analysed the expression of *SERPINA1* in HNC tumour tissue and found a correlation with the expression of genes characteristic for epithelial‐mesenchymal transition (EMT) (negative correlation with epithelial genes *DSP* and *TJP1*, and positive correlation with a set of mesenchymal genes *VIM, FN1, CDH2* and *MMP9*) in HNC (Yu et al. 2024) (Figure 4C).

As Serpin A1 was markedly upregulated in both plasma‐ and tumour cell‐derived sEVs, supporting their potential contribution to neutrophil phenotype reprogramming. Consequently, we investigate the functional consequences of serpin knockout in td‐sEVs releasing cells and its impact on resulting sEV samples, next. Particularly, we compared the function of sEVs of normal and Serpin‐KO cells on the EMT‐activation of sEVs treated neutrophils. Using CRISPR/Cas technology, we generated *SERPINA1* UM‐SCC‐10a knock‐out cells and verified the downregulation of serpin cargo in sEVs produced by this cell line (Figure 4D).

Next, we educated neutrophils using td‐sEVs isolated from both Serpin A1‐deficient (serpin^def^) and Serpin A1‐proficient (serpin^hi^) tumour cell lines, and assessed their phenotypic changes. In agreement with our hypothesis that serpins mediate pro‐tumoural activity of neutrophils, serpin^hi^ sEVs promoted adoption of the pro‐tumour phenotype of neutrophils, characterised by elevated CD11b expression and increased lifespan, while their serpin^def^ sEVs revealed no impact (Figure 4E,F). Next, neutrophils primed with serpin^hi^ versus serpin^def^ sEVs were co‐incubated with tumour cells, and EMT of these cells was assessed. In agreement with the phenotypic changes, serpin^hi^‐stimulated pro‐tumour neutrophils stimulated EMT, as demonstrated by increased density, reduced area (Figure S8) and reduced circularity (Figure 4G,H). Such morphological changes were not observed in cancer cells co‐incubated with neutrophils primed by serpin^def^ sEVs. In agreement with the phenotypic changes, we observed alterations in the expression of EMT markers in tumour cells incubated with such stimulated neutrophils.

The ratio of *VIM* to *TJP1* transcripts (i.e., mesenchymal and epithelial markers, respectively) was also reduced in cancer cells co‐incubated with neutrophils primed by serpin^def^ td‐sEVs, as compared to serpin^hi^ td‐sEVs (Figure 4I). Among the EMT‐associated transcription factors analysed, *TWIST* expression was significantly increased in tumour cells co‐cultured with neutrophils primed by WT (serpin^hi^) td‐sEVs as compared to those primed by A1‐KO (serpin^def^) td‐sEVs (Figure 4J), whereas *SNAIL*, *SLUG*, *ZEB1* and *ZEB2* transcript levels remained unchanged across conditions (Figure S9), indicating that serpin A1‐primed neutrophils selectively engage a *TWIST*‐dependent, rather than broadly pan‐transcriptional, EMT program in tumour cells. This demonstrates that neutrophils primed by sEVs have the potential to efficiently stimulate EMT in cancer cells, and this activity strongly depends on the serpin A1 cargo of td‐sEVs.

### 3.5 Neutrophils Primed by Td‐sEVs Promote T Cell Exhaustion in Serpin‐Dependent Mechanism

In agreement with that, the analysis of transcriptomic GEO database GSE65858^27^ revealed the significant correlation between the expression of *SERPINA1* gene and genes linked to immunosuppressive phenotype (*PDL1, CTLA4, BTLA, IL10, TGFβ2*) in HNC tumour tissue (Figure 5A).

**FIGURE 5:**
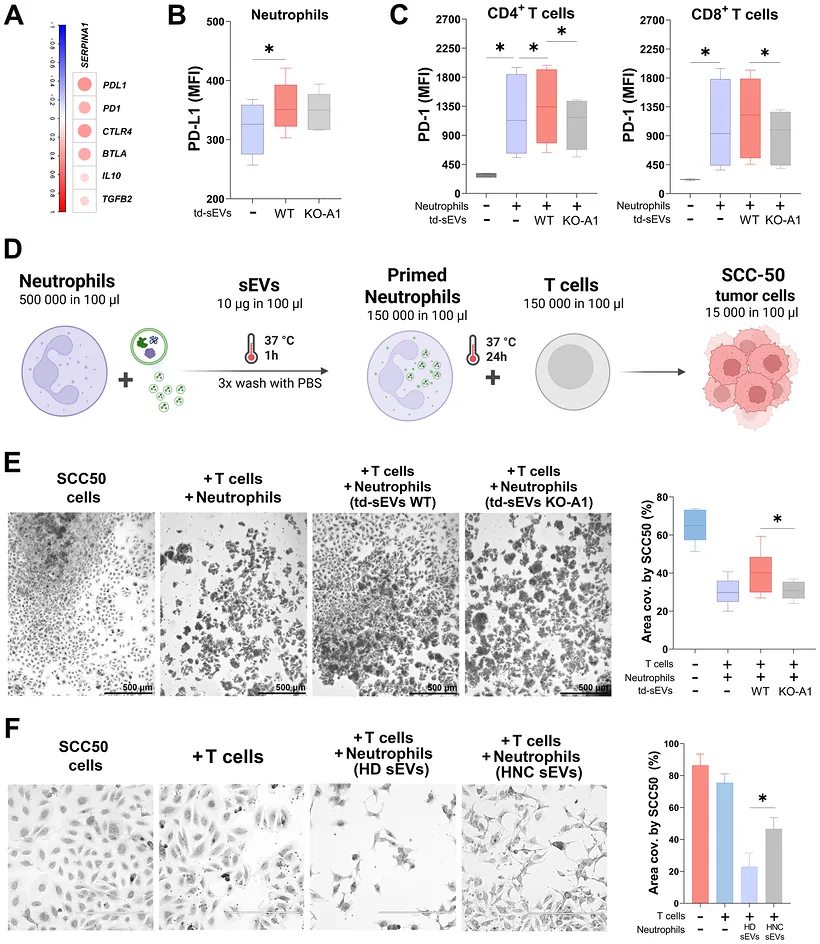
Tumour‐derived sEVs potentiate immunosuppressive features of neutrophils. (A) The correlation between the expression of *SERPIN A1* and the expression of selected genes linked to immunosuppressive phenotype in HNC tumour tissue (analysis was based on the dataset GSE65858^27^). (B) The expression of PD‐L1 was analysed by flow cytometry in neutrophils primed by td‐sEVs released by serpin^hi^ (WT) or serpin^def^ (KO‐A1) UM‐SCC‐10a cells; neutrophils not primed by td‐sEVs are the reference. (C) The expression of PD‐1 was analysed by flow cytometry in T cells (separately for CD4^+^ and CD8^+^ subpopulations) co‐incubated with neutrophils primed by sEVs released by serpin^hi^ or serpin^def^ UM‐SCC‐10a cells; T cells cultured alone or in the presence of neutrophils not primed by td‐sEVs are the reference. (D) Scheme of the experiment: neutrophils primed with sEVs from the UM‐SCC‐10a cells were co‐incubated with autologous T cells, and then co‐cultivated with the UT‐SCC‐50 cells. (E) Morphology and viability of UT‐SCC‐50 cells co‐cultivated with T cells and td‐sEVs primed neutrophils were analysed by light microscopy (cells cultured only in the presence of T cells or T cells pre‐incubated with neutrophils not primed by td‐sEVs are references), shown are representative images, scale bar 500 µm. The graph on the right represents quantitatively the area covered by cancer cells. (F) The morphology and viability of UT‐SCC‐50 cells co‐incubated with T cells and neutrophils primed by sEVs purified from the plasma of healthy donors and HNC patients were analysed by light microscopy (cancer cells cultured alone or only in the presence of T cells are the reference), scale bar 400 µm; the graph on the right represents quantitatively the area covered by cancer cells; * *p* < 0.05. For the comparison of two dependent groups Wilcoxon test was used. Data are shown as bars with a median. Correlations were analysed with the Spearman *R* test. *p < 0.05.

To evaluate the role of sEVs‐associated Serpin A1 in the modulation of the immunosuppressive phenotype of neutrophils, we analysed the expression of PD‐L1 in td‐sEVs primed neutrophils and found it significantly increased upon education with serpin^hi^, but not serpin^def^ td‐sEVs (Figure 5B, D—exp. scheme). Moreover, neutrophils educated by serpin^hi^, but not serpin^def^ td‐sEVs stimulated PD‐1 expression in both CD4^+^ and CD8^+^ T cells (Figure 5C). To functionally address the immunosuppressive activity of T cells after pre‐incubation with td‐sEV‐stimulated neutrophils (Figure 5D), we tested their cytotoxic activity and found significantly elevated cancer cell killing in samples where T cells were stimulated by neutrophils, as compared to T cells alone. However, neutrophils primed by serpin^hi^ sEVs were significantly less efficient than cells stimulated by serpin^def^ sEVs (Figure 5E).

To evaluate whether this phenomenon applies to the situation observed in cancer patients, we analysed tumour‐supportive properties of neutrophils reprogrammed by plasma td‐sEVs. Such neutrophils were co‐incubated with autologous CD8^+^ T cells. Thereafter the cytotoxicity of CD8^+^ cells was assessed. In agreement with our in vitro data, we observed that education of neutrophils with HD plasma sEVs strongly induced the cytotoxic activity of CD8^+^ T cells, in contrast to neutrophils incubated with plasma sEVs of cancer patients (Figure 5F; Figure S10).

This collectively indicates that td‐sEVs education of neutrophils enhances their immunosuppressive phenotype, and that this phenomenon strongly depends on Serpin A1 cargo.

### 3.6 Serpin A1 in sEV Cargo Promotes a Pro‐Tumoural Neutrophil Phenotype by Activating the STAT3/Arginase 1 Axis

To mechanistically dissect the molecular pathway involved in sEV‐mediated neutrophil reprogramming, we assessed proteomic composition of neutrophils after exposure to either serpin^def^ or serpin^hi^ sEVs. Principal component analysis demonstrated significant changes in the neutrophil proteome following education with serpin^hi^ sEVs (Figure 6A). At the same time, depletion of Serpin A1 resulted in a proteomic profile similar to that of naïve, PBS‐treated neutrophils, indicating that serpin cargo is essential for the observed molecular changes in these cells.

**FIGURE 6:**
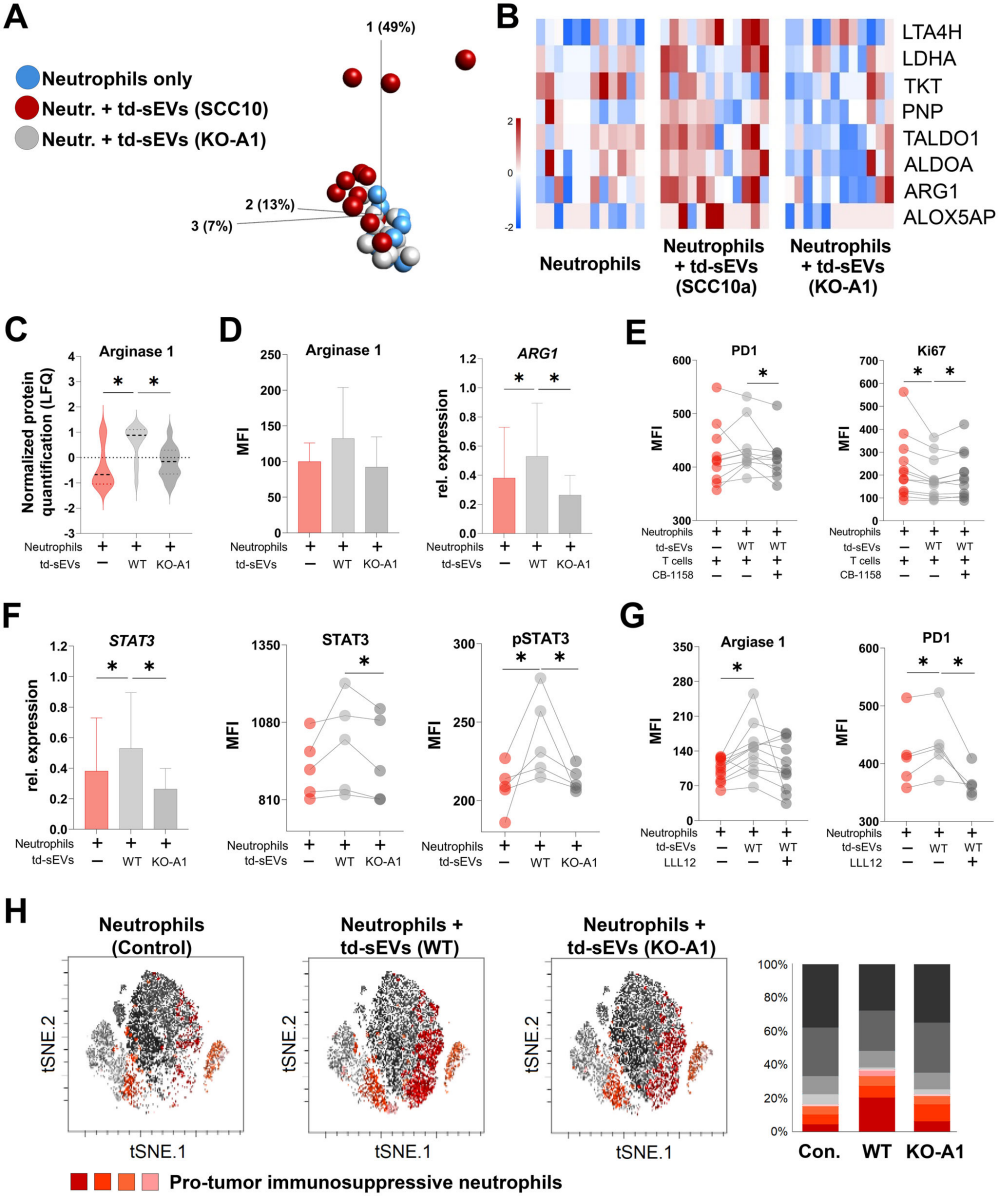
Serpin A1‐containing td‐sEVs promote neutrophil‐mediated immunosuppression via STAT3‐Arginase 1 axis. (A) Principal component analysis (PCA) of neutrophil proteomes following 8‐hour priming with tumour‐derived sEVs enriched in Serpin A1 (red, td‐sEVs SCC10), Serpin A1‐deficient sEVs (grey, td‐sEVs KO‐A1) or PBS (blue, neutrophils only), showing distinct clustering of neutrophils exposed to serpin^hi^ sEVs compared to other groups. (B) Heatmap illustrates protein abundance of selected metabolic and immunosuppressive factors in neutrophils only, neutrophils + serpin^hi^ td‐sEVs (SCC10a), and neutrophils + serpin^def^ td‐sEVs (KO‐A1). (C) Violin plot of normalised label‐free quantification (LFQ) of Arginase 1 protein by MS‐based proteomics, illustrating increased levels in neutrophils primed with serpin^hi^ td‐sEVs versus those with serpin^def^ td‐sEVs or neutrophils alone. (D) Flow cytometry (left) and quantitative PCR (right) for Arginase 1, confirming serpin‐dependent upregulation in neutrophils primed by serpin^hi^ td‐sEVs. (E) T cell co‐culture functional assays show reduced T cell proliferation and enhanced exhaustion after exposure to neutrophils primed with serpin^hi^ td‐sEVs, with functional rescue by Arginase 1 numidargistat. (F) Expression and activation of STAT3 in neutrophils, analysed by qRT‐PCR (STAT3), flow cytometry for total and phosphorylated STAT3 (MFI), demonstrate increased STAT3 activity upon priming with serpin^hi^ td‐sEVs. (G) Functional rescue experiments with STAT3 inhibitor (LLL12). T cells co‐cultured with neutrophils primed with the td‐sEVs following inhibition of STAT3, highlighting restored T cell functions. (H) Multiparametric flow cytometry and tSNE analysis display neutrophil clusters colored according to pro‐tumoural immunosuppressive phenotype, defined by high expression of STAT3 as well as PD‐L1, Arginase 1, pSTAT3, together with activation/immune‐modulatory markers (CD11b, CD66b, IL‐10 and MHC‐1). Bar plots on the right summarise the proportion of neutrophil clusters. For comparison of two dependent groups paired *T* test and Wilcoxon test were used. Data are shown as bars with a median and individual values. *p < 0.05. Panel E: PD‑1 MFI data were collected from three independent experiments, *n* = 10 for each group; Ki‑67 MFI data were collected from five independent experiments, *n* = 12 for each group. Panel F: STAT3 and pSTAT3 MFI data were collected from two independent experiments, *n* = 5 for each group. Panel G: Arginase 1 MFI data were collected from four independent experiments, *n* = 11 for each group; PD‑1 MFI data were collected from two independent experiments, *n* = 10 for each group. STAT3 and ARG1 relative mRNA expression is based on three independent experiments including 14 samples in total.

Pathway enrichment analysis identified significant modulation of the small molecule metabolic process pathway, including Arginase 1, in neutrophils primed with serpin^hi^, but not with serpin^def^ sEVs (Figure 6B, C), suggesting a mechanism of metabolic deprivation that contributes to T cell dysfunction. Flow cytometry and gene expression analysis confirmed that Serpin A1‐dependent priming of donor neutrophils led to increased Arginase 1 transcription and enhanced protein production (Figure 6D).

Next, we assessed the effect of Arginase 1 inhibition on neutrophil‐T cell stimulatory activity. Functionally, pharmacological inhibition of Arginase 1 in neutrophils prevented T cell exhaustion and restored their proliferation, suggesting that serpin A1‐induced neutrophil immunosuppressory activity is Arginase 1‐dependent (Figure 6E).

Since both Arginase 1 and PD‐L1 expression are tightly controlled by the STAT3 activity (Vasquez‐Dunddel et al. 2013; Zerdes et al. 2019), we assessed STAT3 expression in neutrophils upon td‐sEVs stimulation. We demonstrated that serpin^hi^ td‐sEVs promoted *STAT3* gene expression, and enhanced both total and phosphorylated STAT3 protein expression in neutrophils (Figure 6F). Inhibition of serpin^hi^ sEV‐induced phosphorylation of STAT3 in neutrophils using STAT3 inhibitor (LLL12) resulted in a decrease of Arginase 1 expression on neutrophils and significantly improved T cell activation by these cells, as demonstrated by PD‐1 expression (Figure 6G).

Moreover, unsupervised tSNE and X‐shift clustering of multiparameter flow cytometric data revealed distinct neutrophil subpopulations following education with serpin^hi^ versus serpin^def^ sEVs. Neutrophils exposed to serpin^hi^ sEVs showed pronounced expansion of discrete clusters characterised by high expression of pro‐tumour, immunosuppressive phenotype, defined by high expression of STAT3, PD‐L1, Arginase 1 and pSTAT3, together with the activation/immune‐modulatory markers CD11b (high), CD66b (high), IL‐10 (high) and MHC‐I (low), compared to those primed by serpin^def^ sEVs or untreated (PBS‐control) neutrophils (Figure 6H). Quantitative bar plots derived from cluster frequencies confirmed that the percentage of pro‐tumoural immunosuppressive neutrophils was significantly higher in samples treated with serpin^hi^ td‐sEVs, compared to controls and serpin^def^ sEVs conditions (Figure 6H). Marker‐wise tSNE overlays further demonstrated that neutrophil education with serpin^hi^ sEVs resulted in the highest expression of PD‐L1, Arginase 1, pSTAT3 and total STAT3, and a moderate increase of CD11b, CD66b and IL‐10. Notably, clusters enriched in pro‐tumoural markers overlapped with high STAT3 and pSTAT3, confirming that Serpin A1‐enriched td‐sEVs specifically induce STAT3 activation and drive neutrophil polarisation towards a suppressive phenotype (Figure S11).

Treatment of naïve neutrophils with recombinant Serpin A1 alone was sufficient to induce STAT3 phosphorylation, PD‐L1 and Arginase 1 expression, confirming that Serpin A1 itself could serve as a mediator of the major immunosuppression and promote pro‐tumoural neutrophil reprogramming (Figure S12).

Given that Serpin A1 is a physiological inhibitor of neutrophil elastase, an enzyme required for chromatin decondensation during NETosis, we asked whether sEV‐delivered Serpin A1 affects classic neutrophil effector functions, such as reactive oxygen species (ROS) production and NET formation. Therefore, neutrophils were incubated with PBS (control), WT (Serpin A1^hi^) or Serpin A1‐knockout (A1‐KO, Serpin A1^def^) td‐sEVs. ROS production and NET formation were measured under both spontaneous and PMA‐stimulated conditions (Figure S13). No statistically significant differences in ROS production or NET formation were observed. This indicates that sEV‐delivered Serpin A1 does not suppress or alter oxidative burst capacity or NETosis.

### 3.7 Surface and Luminal Serpin A1 Engage Distinct Receptor‐ and Uptake‐Dependent Pathways in Neutrophils

To determine the molecular route by which sEV‐associated Serpin A1 engages neutrophils, we combined biochemical topology mapping with functional receptor‐ and pathway‐blocking experiments. Western blot analysis of serpin A1 in sEVs treated with proteinase K (PrK) alone or in combination with the detergent Triton X‐100 (Tr‐X) revealed a strong serpin A1 signal under control (PBS) conditions, which increased further upon Tr‐X‐mediated membrane permeabilisation, indicating the presence of an additional intracellular/luminal pool of Serpin A1 protected from proteolysis in the intact vesicle. PrK treatment alone reduced the Serpin A1 signal, consistent with efficient removal of a surface‐exposed fraction. Combined PrK and Tr‐X treatment resulted in the lowest signal, reflecting near‐complete degradation of both surface and luminal pools (Figure S14A). To determine the functional relevance of these distinct pools, sEVs pre‐treated with PrK, and neutrophils pre‐treated with an endocytosis inhibitor (Genistein or CK666) or a CD91/LRP1‐blocking antibody, were used in co‐culture assays. PrK‐treated sEVs showed markedly reduced capacity to induce neutrophil viability, CD11b upregulation and STAT3/pSTAT3 activation, mirroring the loss of surface‐accessible Serpin A1 seen by Western blot (Figure S14B). Blockade of CD91/LRP1 similarly reduced sEV‐induced CD11b upregulation and viability, but did not diminish STAT3/pSTAT3 induction (Figure S14C), and inhibition of endocytosis (Genistein/CK666) selectively affected viability and, to a lesser extent, CD11b and STAT3, without affecting PD‐L1 (Figure S14D). Taken together, these results demonstrate that the surface‐exposed pool of Serpin A1 is the primary functional pool driving the acute CD91/LRP1‐dependent activation phenotype (CD11b, viability), whereas the STAT3‐Arginase1‐PD‐L1 axis is engaged through a CD91‐independent, only partially internalisation‐dependent route (Figure S14E).

In summary, our findings demonstrate that Serpin A1 cargo within tumour‐derived sEVs is required to induce tumour‐supportive, immunosuppressive neutrophil subpopulations. Neutrophils exposed to serpin^hi^ sEVs upregulate STAT3 signalling and its targets (PD‐L1 and Arginase 1), whereas no significant reprogramming is observed with serpin^def^ sEVs.

## 4 Discussion

Extracellular vesicles, together with cytokines and growth factors, are well recognised as key regulators of neutrophil function in cancer. In this manuscript, we uncover a previously unknown mechanism whereby tumour‐derived sEVs, through their Serpin A1 cargo, reprogram neutrophils toward a tumour‐supportive and immunosuppressive phenotype. Tumours exploit sEVs as potent mediators of immune suppression by directly inhibiting effector immune cells, modulating immune checkpoint pathways, and restricting lymphocyte infiltration into the tumour microenvironment (Ahmadi et al. 2024). Consistent with this role, tumour‐derived sEVs have been shown to carry multiple immunoregulatory molecules, including PD‐L1, PD‐1, CTLA‐4, FasL, TGF‐β and IL‐10 (Ludwig et al. 2017), which directly influence immune cell function. Here, we identify Serpin A1 as a previously unrecognised component of tumour‐derived sEVs and demonstrate its significant role in driving pro‐tumoural neutrophil reprogramming.

The **SER**ine **P**rotease **IN**hibitor (serpin) superfamily is a diverse group of irreversible protease inhibitors. Despite the significant diversity of serpins, they can be generally grouped according to their structure or functions, for example, inhibitors of coagulation, inhibitors of proteinases, and so forth. Nevertheless, the role of human serpins in a variety of biological processes is not fully understood (Silverman et al. 2001). Serpin A1, also known as alpha‐1‐antitrypsin, belongs to the group of proteinase inhibitors that maintain cellular homeostasis and is crucial for controlling protease activity during inflammation to prevent tissue damage (Janciauskiene et al. 2018; de Mezer et al. 2023). In the context of cancer, protease inhibition plays an important role in supporting tumour cell survival and spread. Increase of serpin A1 levels in plasma has been observed for a large number of malignancies, including hepatocellular carcinoma (Comunale et al. 2010), colorectal cancer (Bujanda et al. 2013), prostate and lung cancer (El‐Akawi et al. 2008), bladder urothelial carcinoma and stomach adenocarcinoma (Kuai et al. 2023). Moreover, upregulation of several types of serpins by the tumour tissue had been previously demonstrated to be associated with an insufficient response to therapy and a poor prognosis (Ibáñez‐Molero et al. 2022). These findings align closely with our data showing a significant increase of serpin A1 cargo in plasma sEVs of HNC patients. In addition, the abundance of serpin A1 in plasma‐derived sEVs correlates with disease severity, supporting its potential utility as a biomarker in HNC.

Tumour cells upregulate the expression of serpins for several reasons, primarily related to enhancing their survival, immune evasion and migration, thus promoting metastasis (Kwon et al. 2014; Valiente et al. 2014; Kwon et al. 2015). Various serpins reprogram immune cells to beconsiscome immunosuppressive. Inhibition of serine proteases by serpins was demonstrated to induce an alternatively activated (M2) macrophage phenotype, which is associated with anti‐inflammatory and tissue repair functions (Xu et al. 2021). In the context of neutrophils, Serpin A1 is known to inhibit neutrophil elastase (NE) (Johnston et al. 2017). In addition, serpins play a pivotal role in inhibiting apoptosis, thereby promoting cell survival (Fehervari 2017). These serpins exert their effects by inhibiting proteases involved in apoptotic signalling pathways, such as granzymes or caspases (Godovikova and Ritchie 2007; Liu et al. 2013), which helps to maintain a reserve of neutrophils in the bone marrow (Baumann et al. 2013).

In our study we demonstrated the immunomodulatory properties of serpin A1 in tumour‐derived sEVs. Besides reducing the toxicity of neutrophil‐derived enzymes (e.g., NE), serpins stabilises long‐lived degranulated (Duerig et al. 2023) neutrophil phenotype and inhibit neutrophil cytotoxicity against tumour cells (Cui et al. 2021). In addition, we observed elevated epithelial‐mesenchymal transition upon stimulation by td‐sEVs primed neutrophils, which was dependent on the presence of serpins.

As we previously demonstrated, neutrophils play a significant role in the regulation of adaptive immune responses in cancer by presenting antigens and activating lymphocytes, or by expressing immune checkpoint molecules such as PD‐L1 and inducing T cell dysfunctional state (Pylaeva et al. 2021, 2022; Hussain et al. 2022). As previously shown by us and others, T‐cell dysfunction is a hallmark of cancer progression and a main avenue of cancer‐induced immunosuppression (Kürten and Ferris 2024). T cell states seem to go through different states of dysfunction, which lie on a continuum (Cillo et al. 2020), with the terminal stage termed ‘exhaustion’ characterised by high expression of PD‐1 as well as multiple other checkpoint receptors, such as CD39, Tim‐3, TIGIT (Kürten et al. 2021). Importantly, activation of dysfunctional T cells is the main treatment modality of currently approved immunotherapy agents in HNC, such as anti‐PD‐1 antibodies (Kürten and Ferris 2024).

Here we suggest a new mechanism of inducing T cell dysfunction: sEVs enriched in Serpin A1 reprogram neutrophils into an immunosuppressive state by activating STAT3/Arginase 1 axis. EV‐derived Serpin A1 robustly induces STAT3 phosphorylation and activation in neutrophils, which results in a strong upregulation of Arginase 1. Importantly, Serpin A1‐depleted sEVs fail to activate this signalling pathway, indicating the critical role of this serpin in this process. This is consistent with data showing that activation of STAT3 in neutrophils drives their reprogramming into a PD‐L1^+^ immunoregulatory neutrophil subset that suppresses T cell responses and promotes tumour progression (Pylaeva et al. 2022). In agreement, Ozel et al. demonstrated recently that neutrophil‐specific STAT3 inhibition reduces tumour growth while expanding cytotoxic CD8^+^ T cells in tumours and draining lymph nodes (Ozel et al. 2025). In line with these observations, pharmacologic or genetic interference with STAT3 signalling in our experimental models reversed sEVs‐mediated upregulation of Arginase 1 in neutrophils and restored T cell effector function, highlighting STAT3 as an important factor linking Serpin A1 to neutrophil‐mediated immunosuppression.

Arginase 1 upregulation provides a complementary mechanism by which sEV‐primed neutrophils mediate both immune suppression and tumour cell plasticity. By depleting extracellular arginine, Arginase 1 limits T cells' proliferation and effector function, contributing to an immunosuppressive tumour microenvironment (Sosnowska et al. 2021). Moreover, Arginase 1 activity has been implicated in EMT and metastasis formation, where neutrophil‐derived arginase and related mediators promote EMT‐associated gene expression and invasive behaviour of colon cancer cells (Wang et al. 2023). In our study, Serpin‐driven Arginase 1 induction in neutrophils led to enhanced EMT in tumour cells and was directly involved in T cell exhaustion.

Our findings demonstrate that a single vesicular cargo protein, serpin A1, can simultaneously engage two mechanistically and topologically distinct signalling routes in the same target cell. While Serpin A1/α1‐antitrypsin has previously been described as a modulator of neutrophil migration, calpain activity and apoptosis through both protease‐inhibitory and non‐inhibitory mechanisms, its role as an EV‐associated ligand engaging a defined cell‐surface receptor (CD91/LRP1) to drive a specific, receptor‐restricted activation program has not, to our knowledge, been previously described. CD91/LRP1 has been characterised mainly as an endocytic receptor for serpin‐enzyme complexes in hepatocytes and macrophages (Congote 2007), and separately as a receptor mediating clearance of apoptotic neutrophils by other phagocytes (Zhao et al. 2024), but its function as a direct receptor for sEV‐delivered serpin cargo on neutrophils themselves is, to our knowledge, a novel finding of this study. Furthermore, by combining biochemical protease‐protection assays with functional receptor and uptake blockade, we show that vesicular Serpin A1 exists as biochemically and functionally separable surface and luminal pools, each preferentially coupled to a distinct downstream neutrophil program: an acute, CD91‐dependent activation/survival response, and a slower, largely CD91‐independent STAT3‐Arginase 1‐PD‐L1 immunosuppressive axis. This dual‐pool, dual‐pathway model adds a new layer of mechanistic resolution to the general concept of EV‐mediated immune cell reprogramming, moving beyond the prevailing view that vesicular cargo acts through a single uptake‐dependent mechanism, and instead demonstrating that a single EV cargo protein can differentially license distinct, parallel functional outputs in the recipient cell depending on its exact vesicular topology and receptor engagement.

As serpin overexpression has been established as both a prognostic and predictive marker across multiple malignancies, they constitute attractive targets for cancer therapy. However, direct pharmacological inhibition of serpins remains challenging. Several miRNAs have been shown to directly interfere with serpin expression, and a limited number of low molecular weight compounds have been reported to inhibit serpin activity or reduce their expression (Soman and Asha Nair 2022). Targeting extracellular vesicles has emerged as a promising therapeutic strategy in cancer, given their important role in intercellular communication and their potential utility as drug delivery vehicles (Wang et al. 2023). Accordingly, multiple approaches have been explored, including inhibition of sEV biogenesis and release from tumour cells (e.g. through inhibition of the endosomal sorting complex required for transport or neutral sphingomyelinase), systemic removal of circulating sEVs using affinity‐based plasmapheresis platforms, and interference with sEV uptake by recipient cells via targeting phosphatidylserine, heparan sulfate proteoglycans, tetraspanins or CD54 (Vader et al. 2014). These strategies have shown encouraging preclinical results.

Our data support a model in which serpin‐proficient tumour‐derived sEVs induce a program of STAT3‐dependent Arginase 1 upregulation in neutrophils that simultaneously suppresses anti‐tumour T cell responses and promotes epithelial–mesenchymal transition. This positions neutrophil STAT3 and Arginase 1 as compelling therapeutic targets to disrupt sEV‐mediated immunosuppression and tumour progression. Such interventions may have important implications for current immunotherapy strategies and could contribute to the development of future combination therapies.

A possible limitation of the study is the age discrepancy between the healthy donor and HNC patient cohorts (mean ages of 38 and 63 years, respectively), which is inherent to the epidemiology of head and neck squamous cell carcinoma. While we cannot entirely exclude a contribution of age‐related inflammaging to baseline neutrophil activation state or to the plasma sEVs proteome, the key mechanistic findings of this study were obtained using healthy (young) donor neutrophils stimulated ex vivo with sEVs, thereby decoupling donor neutrophil age from sEV cargo effects. Furthermore, the stage‐dependent gradient of sEVs serpin A1 abundance observed within the patient cohort—where age is relatively constant—argues against age as the primary driver of the described molecular axis. Nonetheless, future studies using age‐matched healthy controls or employing multivariate models adjusting for age will be needed to fully exclude the contribution of inflammaging to the sEVs proteome and its functional consequences for neutrophil reprogramming.

## 5 Conclusion

In conclusion, our findings collectively highlight a pivotal role of Serpin A1‐enriched tumour‐derived sEVs in neutrophil reprogramming, leading to the establishment of a pro‐tumoural microenvironment that promotes epithelial‐mesenchymal transition, immune evasion and tumour progression. We further propose a new mechanism of neutrophil‐mediated induction of dysfunctional T cells. Understanding these mechanisms provides a conceptual framework for the development of therapeutic strategies aimed at disrupting neutrophil reprogramming in cancer and targeting sEV‐driven immunosuppression.

## Author Contributions

Conceptualization: Maksim Domnich, Ekaterina Pylaeva and Jadwiga Jablonska. Methodology: Maksim Domnich, Ekaterina Pylaeva, Monika Pietrowska, Ilona Thiel, Marta Gawin, Maren Soldierer, Helmut Hanenberg and Jadwiga Jablonska. Software: Maksim Domnich, Ekaterina Pylaeva and Monika Pietrowska. Validation: Maksim Domnich, Ekaterina Pylaeva, Elena Siakaeva and Jadwiga Jablonska. Formal analysis: Maksim Domnich, Ekaterina Pylaeva and Elena Siakaeva. Investigation: Maksim Domnich, Ekaterina Pylaeva, Elena Siakaeva, Nastassia Kabankova, Agnieszka Bedzinska, Damian Sojka, Aneta Zebrowska, Marta Gawin, Maren Soldierer, Malwina Rist, Daniel Fochtman and Monika Pietrowska. Resources: Stephan Lang, Cornelius H. L. Kürten, Monika Pietrowska, Bernd Giebel, Iris Helfrich and Jadwiga Jablonska. Data curation: Maksim Domnich, Ekaterina Pylaeva and Jadwiga Jablonska. Writing – original draft preparation: Maksim Domnich and Ekaterina Pylaeva. Writing – review & editing: Elena Siakaeva, Agnieszka Bedzinska, Damian Sojka, Marta Gawin, Maren Soldierer, Malwina Rist, Daniel Fochtman, Piotr Widlak, Iris Helfrich, Basant Kumar Thakur, Monika Pietrowska, Cornelius H. L. Kürten, Sonja Ludwig and Jadwiga Jablonska. Visualization: Maksim Domnich and Ekaterina Pylaeva. Supervision: Jadwiga Jablonska. Project administration: Jadwiga Jablonska. Funding acquisition: Stephan Lang and Jadwiga Jablonska.

## Conflicts of Interest

The authors declare no conflicts of interest.

## Supporting information




**Supporting Information**: jev270385‐sup‐0001‐TableS1.xlsx

**Supporting Information**: jev270385‐sup‐0001‐TableS2.xlsx

**Supporting Information**: jev270385‐sup‐0001‐TableS3.xlsx

**Supporting Information**: jev270385‐sup‐0001‐TableS4.xlsx

**Supporting Information**: jev270385‐sup‐0005‐FigureS1‐S14.docx

**Supporting Information**: jev270385‐sup‐0006‐SuppMat.xlsx

## Data Availability

The data that support the findings of this study are available from the corresponding author upon reasonable request.
