# Supplementary material for: Serpin A1 Cargo Within Tumour‐Derived Extracellular Vesicles Supports Pro‐Tumoural Bias of Neutrophils in Cancer
Source: J Extracell Vesicles. 2026 Nov 2;15(11):e70385. doi: 10.1002/jev2.70385 (PMC13725528; doi:10.1002/jev2.70385)
Supplement: Supplementary file 1 [file JEV2-15-e70385-s002.docx]

**SUPPLEMENTS**

**Figure S1**

**Purity of isolated neutrophils.** Representative flow cytometry gating strategy for identifying neutrophils in whole blood and isolated neutrophil preparations.

**Figure S2**

**Distribution of study participants according to age and gender.** Plasma samples were collected from 10 healthy individuals (5 males and 5 females, with a mean age of 38 years) and 45 patients (28 males, 60% and 17 females, 40%, with a mean age of 63 years)

**Figure S3**

**The proteome of sEVs isolated from the plasma of HNC patients identifies the regulation of multiple neutrophil-specific signaling pathways.** Plasma-derived sEVs were isolated from individuals with HNC and proceeded for MS-based proteomics. Pathway enrichment analysis of identified proteins is depicted as an enrichment map (p-value < 0.01 and fold change > -1.5, enrichment analysis performed in Enrichment Map app (https://apps.cytoscape.org/apps/enrichmentmap) by using Cytoscape (https://cytoscape.org**/)**. The resulting enrichment map was annotated using AutoAnnotate (https://apps.cytoscape.org/apps/autoannotate) with standard settings with the aim of seeing the general overview of the functions of isolated proteins. In this format, large clusters are no more important than single nodes but it is easy to identify the major themes of analysis.

**Figure S4**

**Nanoparticle tracking analysis** (NTA) of sEVs isolated from tumor HNC-derived cell lines UT-SCC-10a (pink) and UM-SCC50 (blue) revealed similar mean sizes and concentrations. Total protein load of sEVs from advanced cancer stage (UM-SCC-10a) samples was significantly higher in comparison to sEVs from an early stage (UT-SCC-50). For the comparison of two independent groups, the Mann-Whitney *U* test was used. Data are shown as individual values. *** p < 0.005.

**Figure S5**

**Absence of Calnexin in sEVs** confirms the purity of isolated small extracellular vesicles. Western blot analysis for Calnexin (~90 kDa), used as a negative marker for sEV characterization, was performed on whole cell lysates and sEV fractions isolated by size-exclusion chromatography from HNC cell lines UT-SCC-50 (SCC-50) and UM-SCC-10a (SCC-10). Samples in equal amounts (5 µg per well) were loaded.

**Figure S6**

**Gating strategy example. Survival assay**. Neutrophils were gated into distinct populations based on their Annexin (PE) expression level and 7AAD (PerCP-Cy5). Cells characterized by high levels of 7AAD^high^ and Annexin^high^ were classified as dead cells, while those displaying moderate levels of both markers 7AAD^mid^ and Annexin^mid^ were designated as apoptotic. Cell exhibiting low levels of 7AAD^low^ and Annexin^low^ were categorized as alive.

**Figure S7**

**Changes in neutrophil behavior during cancer progression are linked to uptake of td-sEVs, not soluble factors.** In order to rule out the possibility that soluble tumor-derived factors, rather than td-sEVs, are responsible for the observed phenomenon, healthy neutrophils were treated concurrently with both td-sEVs and td-sEVs -depleted tumor-derived supernatants. No significant alterations in marker expression were detected following incubation with td-sEVs -depleted tumor-derived supernatants. Marker’s expression (CD62L, CD11b, CD66b, CXCR2) on neutrophils was estimated by flow cytometry. For comparison of two independent groups, Mann-Whitney U test was used, and for comparison of multiple independent groups Kruskal-Wallis (ANOVA) was used. Data are shown as median with interquartile range and individual values with median. Data are shown as an individual values with median. * p < 0.05, *** p < 0.005.

**Figure S8**

**Quantitative analysis of UT-SCC-50 tumor cell area.** Neutrophils primed with serpin^hi^ versus serpin^def^ sEVs were co-incubated with UT-SCC-50 in the absence or presence of td-sEVs.

**Figure S9**

Relative expression of EMT-associated transcription factors: *TWIST, SNAIL*, *SLUG*, *ZEB1* and *ZEB2* in tumor cells (SCC-10a) co-cultured with neutrophils primed with PBS (control), wild-type (WT) sEVs or Serpin A1-knockout (KO-A1) sEVs. Expression of *SNAIL*, *SLUG*, *ZEB1* and *ZEB2* remained unchanged across conditions.

**Figure S10**

**Direct cell counting confirms robustness of tumor cell quantification independent of methodology.** As an alternative to the cell density-based quantification shown in Figure 5F, UT-SCC-50 tumor cell numbers were additionally assessed by direct cell counting (number of viable adherent tumor cells per field of view, per biological sample) following co-incubation with T cells and neutrophils primed by sEVs purified from the plasma of healthy donors (+HD EVs) or HNC patients (+HNC EVs), with T cells only and SCC50 only as reference conditions. This alternative quantification method reproduced all statistically significant differences reported in Figure 5F (cell density analysis), confirming that neutrophils primed by healthy donor-derived sEVs support significantly greater CD8+ T cell-mediated tumor cell killing compared to those primed by HNC patient-derived sEVs, regardless of the quantification approach used. Data are shown as individual values with mean bars (n=4, 6 fields of view per sample); *p<0.05.

**Figure S11**

**Unbiased clustering reveals STAT3-mediated neutrophil polarization primed by Serpin A1-enriched sEVs.** Neutrophils were isolated from tumor-free mice and cultured in vitro either untreated (PBS), with tumor-derived small extracellular vesicles from wild-type tumor cells (td-sEVs WT), or with Serpin A1-deficient tumor-derived sEVs (td-sEVs KO-A1), followed by multiparametric flow cytometry and tSNE analysis. tSNE maps display neutrophil clusters colored according to pro-tumoral immunosuppressive phenotype, defined by high expression of STAT3 and effector molecules (PD-L1, Arginase 1, pSTAT3, total STAT3) together with activation/immune-modulatory markers (CD11b, CD66b, IL-10), whereas MHC-I remained relatively unchanged across clusters. Bar plots on the right summarize the proportion of neutrophil clusters, illustrating that WT td-sEVs markedly expand pro-tumoral immunosuppressive neutrophil subsets, while KO-A1 td-sEVs largely fail to induce this reprogramming, indicating that Serpin A1-containing td-sEVs are required for STAT3-dependent neutrophil polarization toward a tumor-supportive, immunosuppressive phenotype.

**Figure S12**

**Recombinant Serpin A1 alone is sufficient to induce PD-L1, STAT3, and Arginase 1 expression in neutrophils.** Neutrophils were incubated with PBS or soluble recombinant Serpin A1 (A1) for 18 h, and viability (% alive), CD11b, PD-L1, STAT3, pSTAT3, and Arginase 1 expression were assessed by flow cytometry. Soluble Serpin A1 alone significantly increased PD-L1, STAT3, pSTAT3, and Arginase 1 expression, without significantly affecting neutrophil viability or CD11b expression. Data are shown as individual values with median. For comparison of two independent groups, the Mann-Whitney U test was used. *p < 0.05; ns, not significant.

**Figure S13**

**sEV-Serpin A1 does not significantly affect neutrophil oxidative burst or NET formation.** Neutrophils were incubated with PBS, WT (Serpin A1-sufficient), or A1-KO (Serpin A1-deficient) td-sEVs, and reactive oxygen species (ROS) production and neutrophil extracellular trap (NET) formation (Sytox) were assessed under spontaneous (spont.) and PMA-stimulated (PMA-stim.) conditions. No significant differences were observed between WT and A1-KO conditions for spontaneous or PMA-stimulated ROS production, nor for spontaneous or PMA-stimulated NET formation (Sytox). Data are shown as individual values with median. For comparison between WT and A1-KO groups, the Mann-Whitney U test was used. ns, not significant.

**Figure S14**

**Surface and luminal Serpin A1 engage distinct receptor- and uptake-dependent pathways in neutrophils.** A) Western blot analysis of Serpin A1 (~65 kDa) in td-sEVs treated with Triton X-100 (Tr-X), combined proteinase K and Triton X-100 (PrK+Tr-X), proteinase K (PrK) alone, or PBS (vehicle control). Right panel shows relative Serpin A1 band intensity for each condition, illustrating an additional intracellular/luminal pool of Serpin A1 revealed upon membrane permeabilization (Tr-X), and progressive loss of signal upon proteolytic digestion of surface-exposed (PrK) and, in combination with Tr-X, luminal Serpin A1. (B) Neutrophils were incubated with PBS or proteinase K-treated (PrK) or untreated td-sEVs for 18 h, and viability (% alive), CD11b, PD-L1, STAT3, and pSTAT3 expression were assessed by flow cytometry. (C) Neutrophils were pre-treated with a blocking anti-CD91 antibody (αCD91) or left untreated, prior to incubation with PBS or td-sEVs for 18 h, and viability, CD11b, PD-L1, STAT3, and pSTAT3 expression were assessed by flow cytometry. (D) Neutrophils were pre-treated with the endocytosis inhibitors Genistein (Gen) or CK666, or left untreated, prior to incubation with PBS or td-sEVs for 18 h, and viability, CD11b, PD-L1, STAT3, and pSTAT3 expression were assessed by flow cytometry. (E) Schematic representation of the proposed dual-pathway mechanism, illustrating the surface-exposed, CD91-dependent Serpin A1 pool driving neutrophil activation (CD11b, viability), and the CD91-independent, partially internalization-dependent luminal Serpin A1 pool driving the STAT3-Arginase1-PD-L1 axis. Data are shown as individual values with median. For comparison of multiple groups, Kruskal-Wallis ANOVA with Bonferroni correction was used. *p < 0.05, **p < 0.01; ns, not significant.
